# Supplementary material for: NSC-derived exosomes enhance therapeutic effects of NSC transplantation on cerebral ischemia in mice
Source: eLife. 2023 Apr 27;12:e84493. doi: 10.7554/eLife.84493 (PMC10139690; doi:10.7554/eLife.84493)
Supplement: Figure 1—source data 1. [file elife-84493-fig1-data1.zip › Figure 1-source data 1/Figure 1-source data 1.docx]

**Figure 1C and Figure 1 supplement 2-Resource data: Behavioral data**

| **Balance beam** | **Sham** | | | | | | | | | | | | | | | | | | | | | | | | | | | | | | | | | | | | | | | | | | |  |  |  |  |
| --- | --- | --- | --- | --- | --- | --- | --- | --- | --- | --- | --- | --- | --- | --- | --- | --- | --- | --- | --- | --- | --- | --- | --- | --- | --- | --- | --- | --- | --- | --- | --- | --- | --- | --- | --- | --- | --- | --- | --- | --- | --- | --- | --- | --- | --- | --- | --- |
| 0w | 10.00 | | | 9.50 | | 9.50 | | | | 10.00 | | 9.50 | | | 9.50 | | | | 9.50 | | | | 10.00 | | | | 9.50 | | | | 9.67 | | | | 9.00 | | 9.67 | | | | | | |  |  |  |  |
| 4w | 10.00 | | | 10.00 | | 10.00 | | | | 10.00 | | 9.33 | | | 10.00 | | | | 10.00 | | | | 8.67 | | | | 10.00 | | | | 9.67 | | | | 10.00 | | 9.00 | | | | | | |  |  |  |  |
| 8w | 10.00 | | | 10.00 | | 10.00 | | | | 9.67 | | 9.00 | | | 9.67 | | | | 10.00 | | | | 9.67 | | | | 9.67 | | | | 9.67 | | | | 10.00 | | 9.33 | | | | | | |  |  |  |  |
|  |  | | |  | | |  | | | |  | |  | | | |  | | | |  | | | |  | | | |  | | | |  | |  | | | | |  | | | |  |  |  |  |
| **Balance beam** | **Model** | | | | | | | | | | | | | | | | | | | | | | | | | | | | | | | | | | | | | | | | | | |  |  |  |  |
| 0w | 8.67 | | | 1.67 | | 9.00 | | | | 5.00 | | 5.33 | | | 9.00 | | | | 9.17 | | | | 9.00 | | | | 5.33 | | | | 4.33 | | | | 9.00 | | 8.67 | | | | | | |  | | | |
| 4w | 6.00 | | | 2.67 | | 9.67 | | | | 7.00 | | 6.00 | | | 8.00 | | | | 7.33 | | | | 9.00 | | | | 6.00 | | | | 7.33 | | | | 9.33 | | 8.33 | | | | | | |  | | | |
| 8w | 7.00 | | | 6.67 | | 8.33 | | | | 6.33 | | 7.00 | | | 7.33 | | | | 7.67 | | | | 7.33 | | | | 7.67 | | | | 8.00 | | | | 7.67 | | 7.67 | | | | | | |  | | | |
|  |  | | |  | | |  | | | |  | |  | | | |  | | | |  | | | |  | | | |  | | | |  | |  | | | | |  | | | |  | | | |
| **Balance beam** | **Exo** | | | | | | | | | | | | | | | | | | | | | | | | | | | | | | | | | | | | | | | | | | |  | | | |
| 0w | 7.00 | | | 7.00 | | 6.33 | | | | 8.33 | | 7.00 | | | 4.33 | | | | 6.33 | | | | 9.67 | | | | 7.67 | | | | 8.33 | | | | 8.67 | | 7.67 | | | | | | |  | | | |
| 4w | 8.00 | | | 6.00 | | 5.00 | | | | 8.67 | | 9.67 | | | 7.67 | | | | 5.33 | | | | 9.00 | | | | 9.67 | | | | 5.67 | | | | 6.33 | | 6.67 | | | | | | |  | | | |
| 8w | 8.67 | | | 8.00 | | 8.67 | | | | 8.33 | | 9.00 | | | 8.00 | | | | 6.67 | | | | 8.67 | | | | 8.67 | | | | 8.33 | | | | 8.33 | | 6.67 | | | | | | |  | | | |
|  |  | | |  | | |  | | | |  | |  | | | |  | | | |  | | | |  | | | |  | | | |  | |  | | | | |  | | | |  | | | |
| **Balance beam** | **NSC** | | | | | | | | | | | | | | | | | | | | | | | | | | | | | | | | | |  | | |  | | | | | |  | | | |
| 0w | 6.33 | | | 9.33 | | 8.67 | | | | 7.33 | | 6.33 | | | 5.33 | | | | 5.33 | | | | 5.33 | | | | 8.33 | | | | 8.67 | | | |  | |  | | | | | |  | | |  |  |
| 4w | 8.67 | | | 7.67 | | 9.33 | | | | 9.33 | | 9.33 | | | 9.33 | | | | 8.33 | | | | 8.33 | | | | 8.33 | | | | 8.00 | | | |  | |  | | | | | |  | | |  |  |
| 8w | 9.67 | | | 7.67 | | 9.33 | | | | 9.67 | | 10.00 | | | 8.33 | | | | 8.67 | | | | 9.00 | | | | 9.67 | | | | 7.67 | | | |  | |  | | | | | |  | | |  |  |
|  |  | | |  | | |  | | | |  | |  | | | |  | | | |  | | | |  | | | |  | | | |  | |  | | | | |  | | | |  | | | |
| **Balance beam** | **NSC+Exo** | | | | | | | | | | | | | | | | | | | | | | | | | | | | | | | | | | | | | | | | | | |  | | | |
| 0w | 8.33 | | | 8.67 | | 7.33 | | | | 7.33 | | 9.33 | | | 7.67 | | | | 6.00 | | | | 9.33 | | | | 8.33 | | | | 5.67 | | | | 8.33 | | 3.00 | | | | | | |  | | | |
| 4w | 9.00 | | | 9.00 | | 9.33 | | | | 10.00 | | 9.33 | | | 8.00 | | | | 9.67 | | | | 10.00 | | | | 8.67 | | | | 10.00 | | | | 10.00 | | 9.67 | | | | | | |  | | | |
| 8w | 9.67 | | | 10.00 | | 9.33 | | | | 9.67 | | 9.67 | | | 9.00 | | | | 9.67 | | | | 9.67 | | | | 9.00 | | | | 8.33 | | | | 10.00 | | 10.00 | | | | | | |  | | | |
|  |  | | | | | | | | | | | | | | | | | | | | | | | | | | | | | | | | | | | | | | | | | | |  |  |  |  |
| **Ladder lung** | **Sham** | | | | | | | | | | | | | | | | | | | | | | | | | | | | | | | | | | | | | | | | | | |  |  |  |  |
| 0w | 10.00 | | | 9.50 | | 10.00 | | | | 9.50 | | 10.00 | | | 9.50 | | | | 10.00 | | | | 9.50 | | | | 9.50 | | | | 9.50 | | | | 9.50 | | 9.00 | | | | | | |  |  |  |  |
| 4w | 9.25 | | | 10.00 | | 9.50 | | | | 9.75 | | 10.00 | | | 10.00 | | | | 10.00 | | | | 10.00 | | | | 9.75 | | | | 10.00 | | | | 9.75 | | 10.00 | | | | | | |  |  |  |  |
| 8w | 10.00 | | | 10.00 | | 10.00 | | | | 10.00 | | 10.00 | | | 9.50 | | | | 10.00 | | | | 10.00 | | | | 10.00 | | | | 10.00 | | | | 10.00 | | 10.00 | | | | | | |  |  |  |  |
|  |  |  | | | | | |  | |  | | | |  | | | |  | | | |  | | | |  | | | |  | | | |  |  | | | | | |  | | |  |  |  |  |
| **Ladder lung** | **Model** | | | | | | | | | | | | | | | | | | | | | | | | | | | | | | | | | | | | | | | | | | |  |  |  |  |
| 0w | 7.00 | | | 9.00 | | 8.50 | | | | 8.50 | | 5.00 | | | 7.00 | | | | 3.50 | | | | 6.33 | | | | 6.67 | | | | 8.00 | | | | 7.00 | | 7.50 | | | | | | |  | | | |
| *Continued* | | | | | | | | | | | | | | | | | | | | | | | | | | | | | | | | | | | | | | | | | | | |  | | | |
| 4w | 9.00 | | | 9.50 | | 9.00 | | | | 9.50 | | 7.00 | | | 2.50 | | | | 6.00 | | | | 7.50 | | | | 6.00 | | | | 8.33 | | | | 7.00 | | 7.00 | | | | | | |  | | | |
| 8w | 8.5 | | | 7 | | 7 | | | | 8.5 | | 6.5 | | | 8 | | | | 8 | | | | 8.5 | | | | 8.5 | | | | 9 | | | | 8 | | 8.5 | | | | | | |  | | | |
|  |  |  | | | | | |  | |  | | | |  | | | |  | | | |  | | | |  | | | |  | | | |  |  | | | | | |  | | |  | | | |
| **Ladder lung** | **Exo** | | | | | | | | | | | | | | | | | | | | | | | | | | | | | | | | | | | | | | | | | | |  | | | |
| 0w | 8.50 | | | 6.00 | | 3.00 | | | | 5.00 | | 9.00 | | | 3.00 | | | | 5.50 | | | | 9.00 | | | | 9.00 | | | | 8.00 | | | | 8.50 | | 8.00 | | | | | | |  | | | |
| 4w | 7.67 | | | 9.50 | | 7.00 | | | | 5.33 | | 9.00 | | | 5.33 | | | | 8.50 | | | | 9.50 | | | | 8.33 | | | | 5.00 | | | | 8.00 | | 6.33 | | | | | | |  | | | |
| 8w | 7.33 | | | 8.00 | | 10.00 | | | | 8.00 | | 9.00 | | | 7.00 | | | | 8.00 | | | | 9.50 | | | | 8.50 | | | | 8.00 | | | | 8.00 | | 8.00 | | | | | | |  | | | |
|  |  |  | | | | | |  | |  | | | |  | | | |  | | | |  | | | |  | | | |  | | | |  |  | | | | | |  | | |  | | | |
| **Ladder lung** | **NSC** | | | | | | | | | | | | | | | | | | | | | | | | | | | | | | | | | |  | | | | | |  | | |  | | | |
| 0w | 6.50 | | | 7.00 | | 7.50 | | | | 8.00 | | 9.00 | | | 6.00 | | | | 5.50 | | | | 6.00 | | | | 8.00 | | | | 6.50 | | | |  | |  | | | | |  | | |  |  |  |
| 4w | 7.67 | | | 9.00 | | 9.50 | | | | 10.00 | | 8.00 | | | 10.00 | | | | 8.50 | | | | 8.50 | | | | 8.33 | | | | 10.00 | | | |  | |  | | | | |  | | |  |  |  |
| 8w | 9.50 | | | 10.00 | | 9.50 | | | | 9.00 | | 9.00 | | | 9.50 | | | | 9.50 | | | | 8.50 | | | | 10.00 | | | | 8.50 | | | |  | |  | | | | |  | | |  |  |  |
|  |  |  | | | | | |  | |  | | | |  | | | |  | | | |  | | | |  | | | |  | | | |  |  | | | | | |  | | |  | | | |
| **Ladder lung** | **NSC+Exo** | | | | | | | | | | | | | | | | | | | | | | | | | | | | | | | | | | | | | | | | | | |  | | | |
| 0w | 7.50 | | | 8.50 | | 6.00 | | | | 7.50 | | 9.00 | | | 6.50 | | | | 5.50 | | | | 6.50 | | | | 9.50 | | | | 3.50 | | | | 7.00 | 6.00 | | | | | | | |  | | | |
| 4w | 10.00 | | | 9.50 | | 8.50 | | | | 9.50 | | 10.00 | | | 10.00 | | | | 7.67 | | | | 9.50 | | | | 10.00 | | | | 10.00 | | | | 10.00 | 9.50 | | | | | | | |  | | | |
| 8w | 9.00 | | | 10.00 | | 9.00 | | | | 10.00 | | 9.50 | | | 10.00 | | | | 9.00 | | | | 10.00 | | | | 10.00 | | | | 9.00 | | | | 10.00 | 10.00 | | | | | | | |  | | | |
|  |  | | | | | | | | | | | | | | | | | | | | | | | | | | | | | | | | | | | | | | | | | | |  | | | |
| **Rotarod test** | **Sham** | | | | | | | | | | | | | | | | | | | | | | | | | | | | | | | | | | | | | | | | | | |  | | | |
| 0w | 300.00 | | | 300.00 | | 300.00 | | | | 300.00 | | 300.00 | | | 300.00 | | | | 300.00 | | | | 300.00 | | | | 300.00 | | | | 260.00 | | | | 300.00 | 300.00 | | | | | | | |  | | | |
| 4w | 300.00 | | | 300.00 | | 300.00 | | | | 300.00 | | 274.17 | | | 295.00 | | | | 288.33 | | | | 300.00 | | | | 300.00 | | | | 270.67 | | | | 295.00 | 300.00 | | | | | | | |  | | | |
| 8w | 300.00 | | | 293.33 | | 300.00 | | | | 300.00 | | 291.39 | | | 277.22 | | | | 296.11 | | | | 300.00 | | | | 300.00 | | | | 290.22 | | | | 298.33 | 300.00 | | | | | | | |  | | | |
|  |  | |  | |  | | | |  | | |  | | | |  | | | |  | | | |  | | | |  | | | |  | | |  | | | |  | | | | |  | | | |
| **Rotarod test** | **Model** | | | | | | | | | | | | | | | | | | | | | | | | | | | | | | | | | | | | | | | | | | |  |  |  |  |
| 0w | 153.00 | | | 151.00 | | 224.67 | | | | 54.00 | | 251.00 | | | 48.33 | | | | 300.00 | | | | 300.00 | | | | 261.33 | | | | 42.00 | | | | 239.33 | 300.00 | | | | | | | | 224.00 | | |  |
| 4w | 151.33 | | | 36.33 | | 300.00 | | | | 300.00 | | 52.33 | | | 110.33 | | | | 4.00 | | | | 39.33 | | | | 43.67 | | | | 300.00 | | | | 52.00 | 48.33 | | | | | | | | 243.33 | | |  |
| 8w | 300.00 | | | 126.33 | | 300.00 | | | | 300.00 | | 35.00 | | | 11.00 | | | | 42.67 | | | | 228.33 | | | | 41.33 | | | | 292.00 | | | | 71.00 | 129.33 | | | | | | | | 129.00 | | |  |
|  |  | |  | |  | | | |  | | |  | | | |  | | | |  | | | |  | | | |  | | | |  | | |  | |  | | | | | | |  | | | |
| **Rotarod test** | **Exo** | | | | | | | | | | | | | | | | | | | | | | | | | | | | | | | | | | | | | | | | | | |  | | | |
| 0w | 263.00 | | | 245.00 | | 24.67 | | | | 25.67 | | 100.33 | | | 48.33 | | | | 72.67 | | | | 300.00 | | | | 300.00 | | | | 241.67 | | | | 300.00 | 286.33 | | | | | | | |  | | | |
| 4w | 279.11 | | | 11.67 | | 216.00 | | | | 192.56 | | 104.67 | | | 6.33 | | | | 20.67 | | | | 300.00 | | | | 288.00 | | | | 8.33 | | | | 256.50 | 109.17 | | | | | | | |  | | | |
| 8w | 270.21 | | | 120.67 | | 204.78 | | | | 215.85 | | 103.12 | | | 5.86 | | | | 241.24 | | | | 56.67 | | | | 287.30 | | | | 300.00 | | | | 253.56 | 100.90 | | | | | | | |  | | | |

| **Rotarod test** | | **NSC** | | | | | | | | | | | | | | | | | | | | | | | | | | | | | | | | |  | | |  | | | |  | | | |  |  |
| --- | --- | --- | --- | --- | --- | --- | --- | --- | --- | --- | --- | --- | --- | --- | --- | --- | --- | --- | --- | --- | --- | --- | --- | --- | --- | --- | --- | --- | --- | --- | --- | --- | --- | --- | --- | --- | --- | --- | --- | --- | --- | --- | --- | --- | --- | --- | --- |
| 0w | 300.00 | | 141.00 | | | | 120.00 | | | | 113.00 | | | | 124.33 | | 300.00 | | | 130.00 | | | | 241.00 | | | | 225.00 | | | 300.00 | | | |  |  | | | | |  | | |  |  |  |  |
| 4w | 212.67 | | 249.33 | | | | 292.67 | | | | 172.67 | | | | 300.00 | | 300.00 | | | 122.67 | | | | 300.00 | | | | 45.00 | | | 65.33 | | | |  |  | | | | |  | | |  |  |  |  |
| 8w | 12.00 | | 300.00 | | | | 216.67 | | | | 171.67 | | | | 300.00 | | 300.00 | | | 173.67 | | | | 79.00 | | | | 300.00 | | | 290.00 | | | |  |  | | | | |  | | |  |  |  |  |
|  | |  | |  | | | |  | | | |  | | | |  | | |  | | | |  | | | |  | | |  | | | |  |  | | |  | | | |  | | | |  |  |
| **Rotarod test** | | **NSC+Exo** | | | | | | | | | | | | | | | | | | | | | | | | | | | | | | | | | | | | | | | |  | | | |  |  |
| 0w | | 261.33 | | | 217.33 | | | | 224.00 | | | | 179.67 | | | 188.33 | | 300.00 | | | | 23.67 | | | | 300.00 | | | 300.00 | | | 122.67 | | | 122.00 | | 53.00 | | | | |  | | | |  |  |
| 4w | | 300.00 | | | 46.67 | | | | 300.00 | | | | 153.33 | | | 259.33 | | 300.00 | | | | 69.67 | | | | 239.00 | | | 247.67 | | | 198.00 | | | 268.67 | | 300.00 | | | | |  | | | |  |  |
| 8w | | 300.00 | | | 65.00 | | | | 300.00 | | | | 300.00 | | | 239.67 | | 274.33 | | | | 98.67 | | | | 300.00 | | | 236.67 | | | 260.00 | | | 233.00 | | 300.00 | | | | |  | | | |  |  |
|  | |  | | |  | | | |  | | | |  | | |  | |  | | | |  | | | |  | | |  | | |  | | |  | |  | | | | |  | | | |  |  |
| **mNss** | | **Sham** | | | | | | | | | | | | | | | | | | | | | | | | | | | | | | | | | | | | | | | |  | | | |  |  |
| 0w | | 0 | | | 0 | | | | 0 | | | | 0 | | | 1 | | 0 | | | | 0 | | | | 0 | | | 0 | | | 0 | | | 0 | | 0 | | | | |  | | | |  |  |
| 4w | | 0 | | | 0 | | | | 0 | | | | 0 | | | 0 | | 0 | | | | 0 | | | | 1 | | | 0 | | | 0 | | | 0 | | 0 | | | | |  | | | |  |  |
| 8w | | 0 | | | 0 | | | | 0 | | | | 0 | | | 1 | | 2 | | | | 0 | | | | 0 | | | 0 | | | 0 | | | 0 | | 0 | | | | |  | | | |  |  |
|  | |  | |  | |  | | | |  | | | |  | | |  | | | |  | | | |  | | | |  | | | |  | |  | | | |  | | |  | | | | |  |
| **mNss** | | **Model** | | | | | | | | | | | | | | | | | | | | | | | | | | | | | | | | | | | | | | | |  |  |  |  |  |  |
| 0w | | 6 | | | 9 | | | | 4 | | | | 9 | | | 9 | | 6 | | | | 5 | | | | 8 | | | 8 | | | 9 | | | 6 | | 9 | | | | | 3 | | |  |  |  |
| 4w | | 4 | | | 8 | | | | 5 | | | | 9 | | | 8 | | 7 | | | | 3 | | | | 5 | | | 6 | | | 7 | | | 4 | | 8 | | | | | 4 | | |  |  |  |
| 8w | | 3 | | | 6 | | | | 4 | | | | 8 | | | 7 | | 5 | | | | 3 | | | | 5 | | | 5 | | | 4 | | | 3 | | 4 | | | | | 4 | | |  |  |  |
|  | |  | |  | |  | | | |  | | | |  | | |  | | | |  | | | |  | | | |  | | | |  | |  | | |  | | | |  | | | | |  |
| **mNss** | | **Exo** | | | | | | | | | | | | | | | | | | | | | | | | | | | | | | | | | | | | | | | |  | | | |  |  |
| 0w | | 6 | | | 7 | | | | 9 | | | | 7 | | | 8 | | 9 | | | | 9 | | | | 5 | | | 5 | | | 3 | | | 3 | | 10 | | | | |  | | | | |  |
| 4w | | 5 | | | 4 | | | | 6 | | | | 5 | | | 4 | | 8 | | | | 9 | | | | 3 | | | 4 | | | 8 | | | 6 | | 6 | | | | |  | | | | |  |
| 8w | | 3 | | | 4 | | | | 3 | | | | 3 | | | 4 | | 6 | | | | 8 | | | | 3 | | | 3 | | | 8 | | | 5 | | 4 | | | | |  | | | | |  |
|  | |  | |  | | | |  | |  | | | |  | | |  | | | |  | | | |  | | | |  | | | |  | |  | | |  | | | |  | | | | |  |
| **mNss** | | **NSC** | | | | | | | | | | | | | | | | | | | | | | | | | | | | | | | | |  | | |  | | | |  | | | |  |  |
| 0w | | 7 | | | 6 | | | | 3 | | | | 9 | | | 7 | | 7 | | | | 9 | | | | 6 | | | 7 | | | 6 | | |  | | | | |  | | |  | | | | |
| 4w | | 3 | | | 3 | | | | 2 | | | | 3 | | | 2 | | 4 | | | | 5 | | | | 3 | | | 2 | | | 2 | | |  | | | | |  | | |  | | | | |
| 8w | | 6 | | | 0 | | | | 2 | | | | 1 | | | 2 | | 1 | | | | 3 | | | | 3 | | | 0 | | | 2 | | |  | | | | |  | | |  | | | | |
|  | |  | |  | | | |  | |  | | | |  | | |  | | | |  | | | |  | | | |  | | | |  | |  | | |  | | | |  | | | | |  |
| **mNss** | | **NSC+Exo** | | | | | | | | | | | | | | | | | | | | | | | | | | | | | | | | | | | | | | | |  | | | |  |  |
| 0w | | 7 | | | 6 | | | | 7 | | | | 9 | | | 7 | | 7 | | | | 9 | | | | 6 | | | 7 | | | 8 | | | 7 | | 7 | | | | |  | | | | |  |
| 4w | | 2 | | | 3 | | | | 1 | | | | 3 | | | 1 | | 1 | | | | 4 | | | | 1 | | | 1 | | | 5 | | | 1 | | 1 | | | | |  | | | | |  |
| 8w | | 1 | | | 2 | | | | 0 | | | | 0 | | | 1 | | 2 | | | | 2 | | | | 0 | | | 0 | | | 2 | | | 1 | | 0 | | | | |  | | | | |  |

| **Figure 1E-Resource data:** **Infract volume data** | | | | | | | | | | |
| --- | --- | --- | --- | --- | --- | --- | --- | --- | --- | --- |
|  | Ipsilesional | | | Average | Contralesional | | | Average | Ips/Con | |
| M-1 | 422.00 | 347.00 | 327.00 | 365.33 | 2944.00 | 2760.00 | 2440.00 | 2714.67 |  | |
|  | 1224.00 | 1218.00 | 968.00 | 1136.67 | 3367.00 | 3285.00 | 3461.00 | 3371.00 |  | |
|  | 839.00 | 734.00 | 543.00 | 705.33 | 2434.00 | 2327.00 | 2234.00 | 2331.67 |  | |
|  |  |  |  | 735.78 |  |  |  | 2805.78 | 0.26 | |
| M-2 | 135.00 | 91.00 | 101.00 | 109.00 | 1865.00 | 2058.00 | 1959.00 | 1960.67 |  | |
|  | 562.00 | 486.00 | 529.00 | 525.67 | 2634.00 | 2481.00 | 2361.00 | 2492.00 |  | |
|  | 174.00 | 173.00 | 175.00 | 174.00 | 2918.00 | 2765.00 | 2714.00 | 2799.00 |  | |
|  |  |  |  | 808.67 |  |  |  | 7251.67 | 0.11 | |
| M-3 | 328.00 | 334.00 | 301.00 | 321.00 | 2740.00 | 2945.00 | 2623.00 | 2769.33 |  | |
|  | 264.00 | 258.00 | 277.00 | 266.33 | 2989.00 | 3078.00 | 3049.00 | 3038.67 |  | |
|  | 124.00 | 116.00 | 123.00 | 121.00 | 2853.00 | 2457.00 | 2441.00 | 2583.67 |  | |
|  |  |  |  | 708.33 |  |  |  | 8391.67 | 0.08 | |
| E-1 | 369.00 | 313.00 | 346.00 | 342.67 | 2283.00 | 2182.00 | 2219.00 | 2228.00 |  | |
|  | 482.00 | 532.00 | 490.00 | 501.33 | 2828.00 | 2463.00 | 2543.00 | 2611.33 |  | |
|  | 190.00 | 185.00 | 178.00 | 184.33 | 2892.00 | 2698.00 | 2476.00 | 2688.67 |  | |
|  |  |  |  | 1028.33 |  |  |  | 7528.00 | 0.14 | |
| E-2 | 302.00 | 327.00 | 339.00 | 322.67 | 2291.00 | 2327.00 | 2230.00 | 2282.67 |  | |
|  | 483.00 | 470.00 | 508.00 | 487.00 | 2943.00 | 2785.00 | 2820.00 | 2849.33 |  | |
|  | 276.00 | 165.00 | 149.00 | 196.67 | 3154.00 | 3146.00 | 2866.00 | 3055.33 |  | |
|  |  |  |  | 1006.33 |  |  |  | 8187.33 | 0.12 | |
| E-3 | 512.00 | 513.00 | 441.00 | 488.67 | 2388.00 | 2562.00 | 2546.00 | 2498.67 |  | |
|  | 1317.00 | 1317.00 | 1261.00 | 1298.33 | 3274.00 | 3167.00 | 2863.00 | 3101.33 |  | |
|  | 1042.00 | 1018.00 | 997.00 | 1019.00 | 3322.00 | 3053.00 | 3106.00 | 3160.33 |  | |
|  |  |  |  | 2806.00 |  |  | 8515.00 |  | 0.33 | |
| N-1 | 150.00 | 147.00 | 115.00 | 137.33 | 2707.00 | 2948.00 | 2911.00 | 2855.33 |  | |
|  | 125.00 | 87.00 | 90.00 | 100.67 | 3092.00 | 2930.00 | 2942.00 | 2988.00 |  | |
|  |  |  |  | 238.00 |  |  |  | 5843.33 | 0.04 | |
| N-2 | 268.00 | 257.00 | 293.00 | 272.67 | 2502.00 | 2630.00 | 2595.00 | 2575.67 |  | |
|  | 322.00 | 266.00 | 224.00 | 270.67 | 2638.00 | 2621.00 | 2763.00 | 2674.00 |  | |
|  | 113.00 | 77.00 | 63.00 | 84.33 | 2680.00 | 2670.00 | 2550.00 | 2633.33 |  | |
|  |  |  |  | 627.67 |  |  |  | 7883.00 | 0.08 | |
| N-3 | 67.00 | 70.00 | 50.00 | 62.33 | 2812.00 | 2700.00 | 2724.00 | 2745.33 |  | |
|  | 149.00 | 125.00 | 125.00 | 133.00 | 3084.00 | 2789.00 | 2630.00 | 2834.33 |  | |
|  |  |  |  | 195.33 |  |  |  | 5579.67 | 0.04 | |
| NE-1 | 67.00 | 67.00 | 46.00 | 60.00 | 2953.00 | 2952.00 | 2765.00 | 2890.00 |  | |
| *Continued* | | | | | | | | | | |
|  | 68.00 | 67.00 | 63.00 | 66.00 | 2975.00 | 2591.00 | 2876.00 | 2814.00 |  | |
|  |  |  |  | 126.00 |  |  |  | 5704.00 | 0.02 | |
| NE-2 | 97.00 | 83.00 | 70.00 | 83.33 | 2796.00 | 2724.00 | 2515.00 | 2678.33 |  | |
|  | 93.00 | 77.00 | 172.00 | 114.00 | 2549.00 | 2524.00 | 2701.00 | 2591.33 |  | |
|  |  |  |  | 197.33 |  |  |  | 5269.67 | 0.04 | |
| NE-3 | 592.00 | 623.00 | 647.00 | 620.67 | 2574.00 | 2523.00 | 2435.00 | 2510.67 |  | |
|  | 407.00 | 427.00 | 378.00 | 404.00 | 3229.00 | 3115.00 | 2851.00 | 3065.00 |  | |
|  |  |  |  | 1024.67 |  |  |  | 5575.67 | 0.18 | |
| **Figure 1G-Resource data: Brain weight data** | | | | | | | | | | |

| **group** | **label** | **weight(g)** |
| --- | --- | --- |
| sham | S98 | 0.36 |
|  | S96 | 0.34 |
|  | S99 | 0.35 |
|  | S95 | 0.36 |
|  | S93 | 0.35 |
|  | S92 | 0.37 |
| Model | M5 | 0.31 |
|  | M3 | 0.27 |
|  | M12 | 0.29 |
|  | M4 | 0.30 |
|  | M9 | 0.32 |
| EXO | E43 | 0.33 |
|  | E45 | 0.31 |
|  | E38 | 0.32 |
|  | E47 | 0.31 |
|  | E37 | 0.32 |
| NSC | N22 | 0.32 |
|  | N53 | 0.33 |
|  | N51 | 0.31 |
|  | N24 | 0.32 |
|  | N25 | 0.35 |
|  | N50 | 0.34 |
| NE | NE63 | 0.33 |
|  | NE68 | 0.36 |
|  | NE66 | 0.32 |
|  | NE65 | 0.34 |
|  | NE61 | 0.37 |
|  | NE70 | 0.32 |
