# Supplementary material for: NSC-derived exosomes enhance therapeutic effects of NSC transplantation on cerebral ischemia in mice
Source: eLife. 2023 Apr 27;12:e84493. doi: 10.7554/eLife.84493 (PMC10139690; doi:10.7554/eLife.84493)
Supplement: Figure 1—figure supplement 1—source data 1. [file elife-84493-fig1-figsupp1-data1.zip › Figure 1 supplement 1-source data 1/Figure 1 supplement 1-source data 1.docx]

**Figure 1 supplement 2F-Resource data: Survival rate:**

| Days elapsed | Sham | Model | 2x10^5^ | 5x10^5^ |
| --- | --- | --- | --- | --- |
| 9 | 0 |  | 1 |  |
| 7 | 0 |  |  |  |
| 9 | 0 | 1 |  |  |
| 35 | 0 | 0 |  |  |
| 35 | 0 | 0 |  |  |
| 35 | 0 | 0 |  |  |
| 35 | 0 | 0 |  |  |
| 35 | 0 | 0 |  |  |
| 35 | 0 | 0 |  |  |
| 35 | 0 |  |  |  |
| 35 |  |  | 0 | 0 |
| 35 |  |  | 0 | 0 |
| 35 |  |  | 0 | 0 |
| 35 |  |  | 0 | 0 |
| 35 |  |  | 0 | 0 |
| 35 |  |  |  | 0 |
| 4 |  |  | 1 |  |
| 4 |  | 1 | 1 |  |
| 6 |  |  |  |  |
| 6 |  |  |  |  |
| 6 |  |  |  | 1 |
| 6 |  |  |  | 1 |
| 5 |  | 1 |  | 1 |
| 5 |  | 1 |  | 1 |
| 9 |  | 1 |  | 0 |
| 9 |  | 1 |  | 0 |
| 9 |  | 1 |  | 0 |
| 9 |  | 1 |  | 0 |

**Figure 1 supplement 2G-Resource data: Latency to fall:**

|  | Sham | | | | | | |
| --- | --- | --- | --- | --- | --- | --- | --- |
| 0w | 300 | 234.33 | 300 | 300 | 240 | 300 | 300 |
| 2w | 300 | 220 | 300 | 283.67 | 241.33 | 300 | 300 |
| 4w | 234 | 300 | 300 | 300 | 230 | 300 | 210 |

|  | Model | | | | | | | | |
| --- | --- | --- | --- | --- | --- | --- | --- | --- | --- |
| 0w | 90 | 240 | 22 | 101.67 | 109.67 | 300 | 144.67 | 13.67 | 95.67 |
| 2w | 20 | 19 | 35.67 | 58.33 | 99 | 96.67 | 50 | 40.67 | 44 |
| 4w | 30 | 11.67 | 60 | 30 | 29 | 59 | 27.67 | 40 | 40 |

|  | 2x10^5^ | | | | |
| --- | --- | --- | --- | --- | --- |
| 0w | 141.33 | 100 | 72.67 | 203 | 163.33 |
| 2w | 112 | 160 | 120 | 70.33 | 70 |
| 4w | 89 | 100 | 108 | 123.67 | 111 |

|  | 5x10^5^ | | | | | |
| --- | --- | --- | --- | --- | --- | --- |
| 0w | 120 | 130 | 158 | 150 | 70 | 150 |
| 2w | 300 | 65 | 122.33 | 120 | 135 | 166.67 |
| 4w | 212.67 | 249.33 | 292.67 | 172.67 | 300 | 300 |
