# Supplementary figures and images for: NSC-derived exosomes enhance therapeutic effects of NSC transplantation on cerebral ischemia in mice
Source: eLife. 2023 Apr 27;12:e84493. doi: 10.7554/eLife.84493 (PMC10139690; doi:10.7554/eLife.84493)

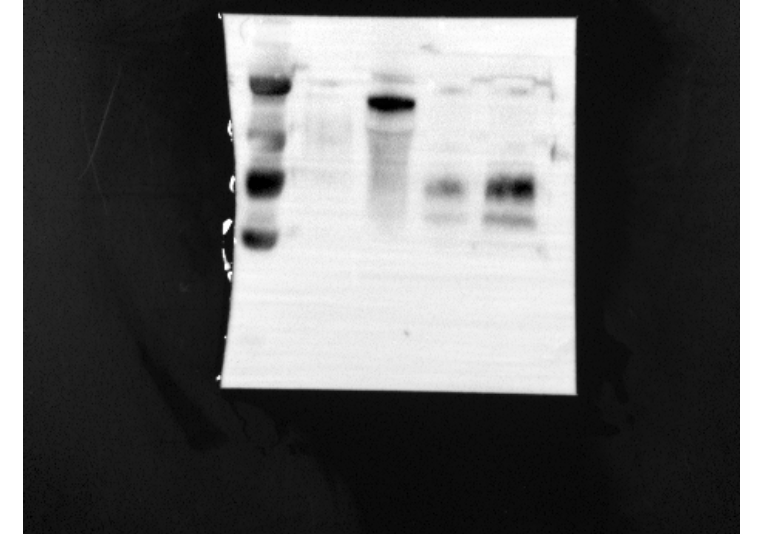

Supplement: Figure 1—figure supplement 1—source data 2. [file elife-84493-fig1-figsupp1-data2.zip › Figure 1 supplement 1-source data 2/The original files /cd63 2020-08-28 12 μù╢ 55 σêå_Exposure_28.3sec.tif]

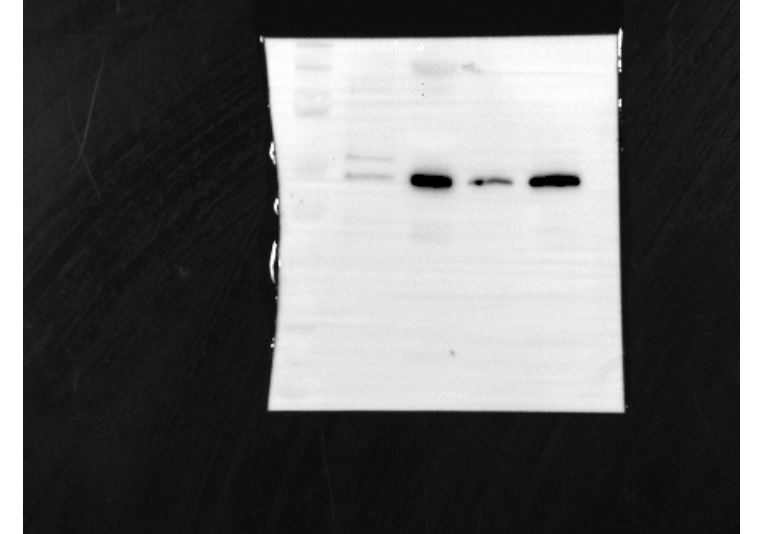

Supplement: Figure 1—figure supplement 1—source data 2. [file elife-84493-fig1-figsupp1-data2.zip › Figure 1 supplement 1-source data 2/The original files /tsg101 2020-08-27 12 μù╢ 45 σêå_Exposure_37.4sec.tif]

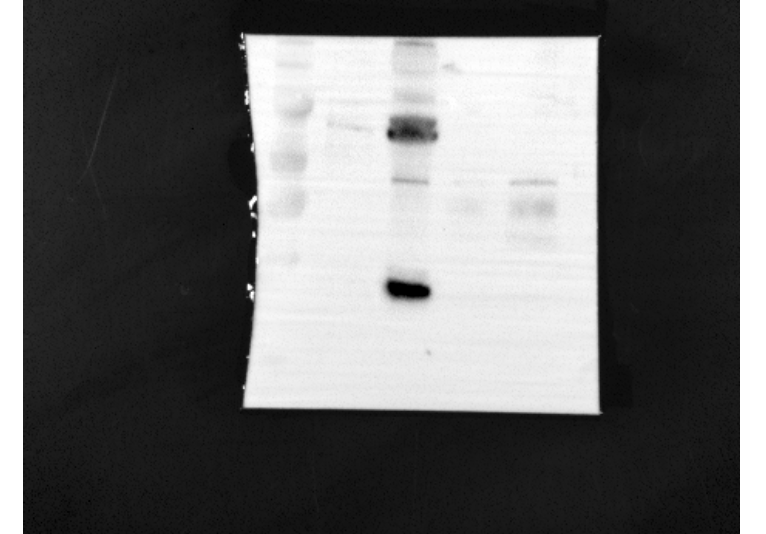

Supplement: Figure 1—figure supplement 1—source data 2. [file elife-84493-fig1-figsupp1-data2.zip › Figure 1 supplement 1-source data 2/The original files /cd9.tif]

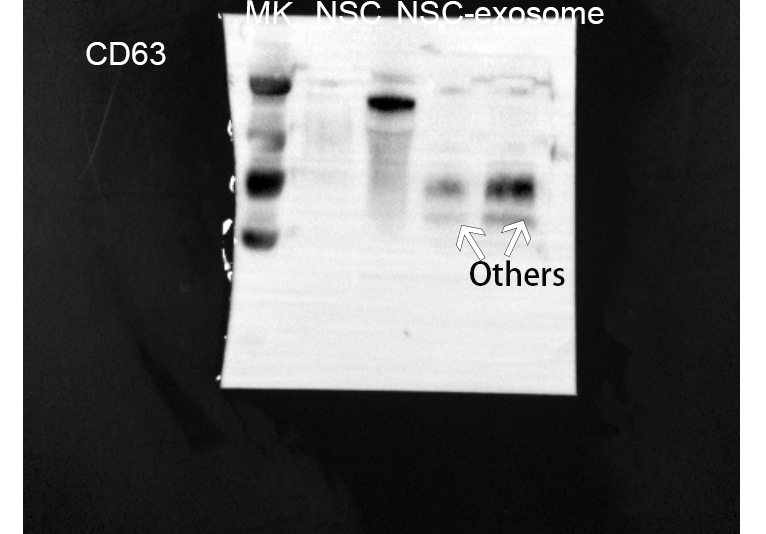

Supplement: Figure 1—figure supplement 1—source data 2. [file elife-84493-fig1-figsupp1-data2.zip › Figure 1 supplement 1-source data 2/The labeled files/cd63 2020-08-28 12 hour 55 minutes_Exposure_28.3sec.tif]

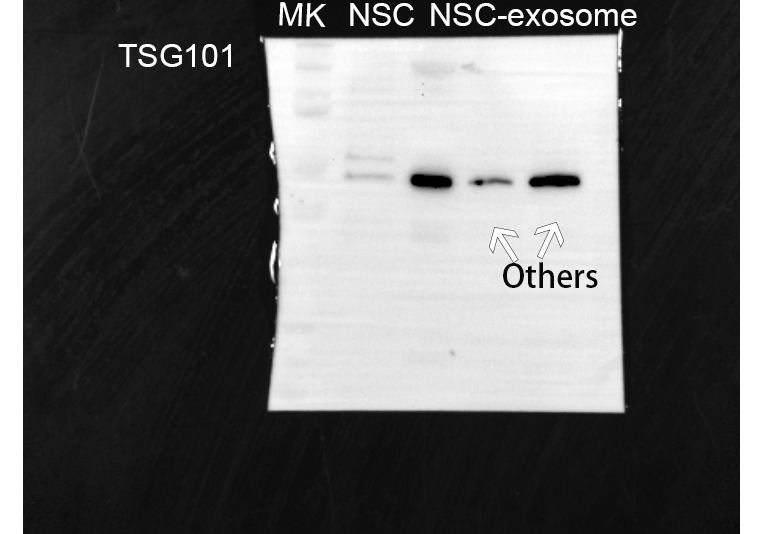

Supplement: Figure 1—figure supplement 1—source data 2. [file elife-84493-fig1-figsupp1-data2.zip › Figure 1 supplement 1-source data 2/The labeled files/tsg101 2020-08-27 12 hour 45 minutes_Exposure_37.4sec.tif]

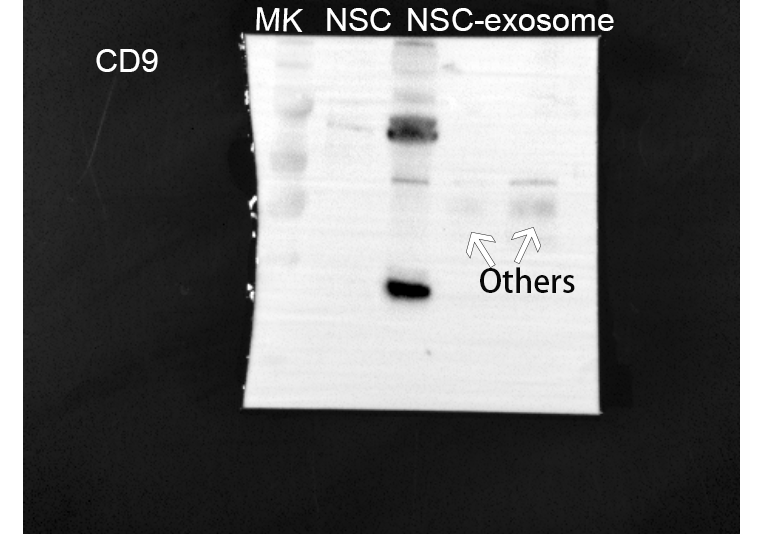

Supplement: Figure 1—figure supplement 1—source data 2. [file elife-84493-fig1-figsupp1-data2.zip › Figure 1 supplement 1-source data 2/The labeled files/cd9.tif]

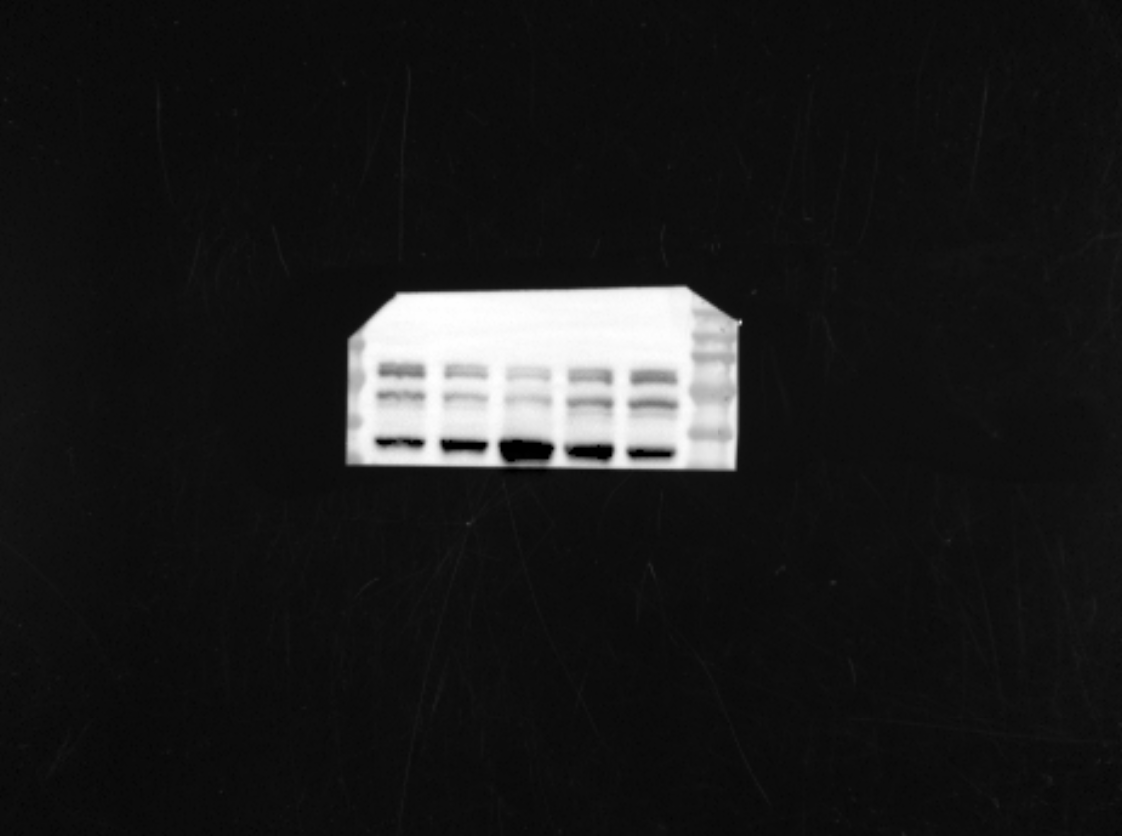

Supplement: Figure 2—source data 1. [file elife-84493-fig2-data1.zip › Figure 2-source data 1/The original files /syn.tif]

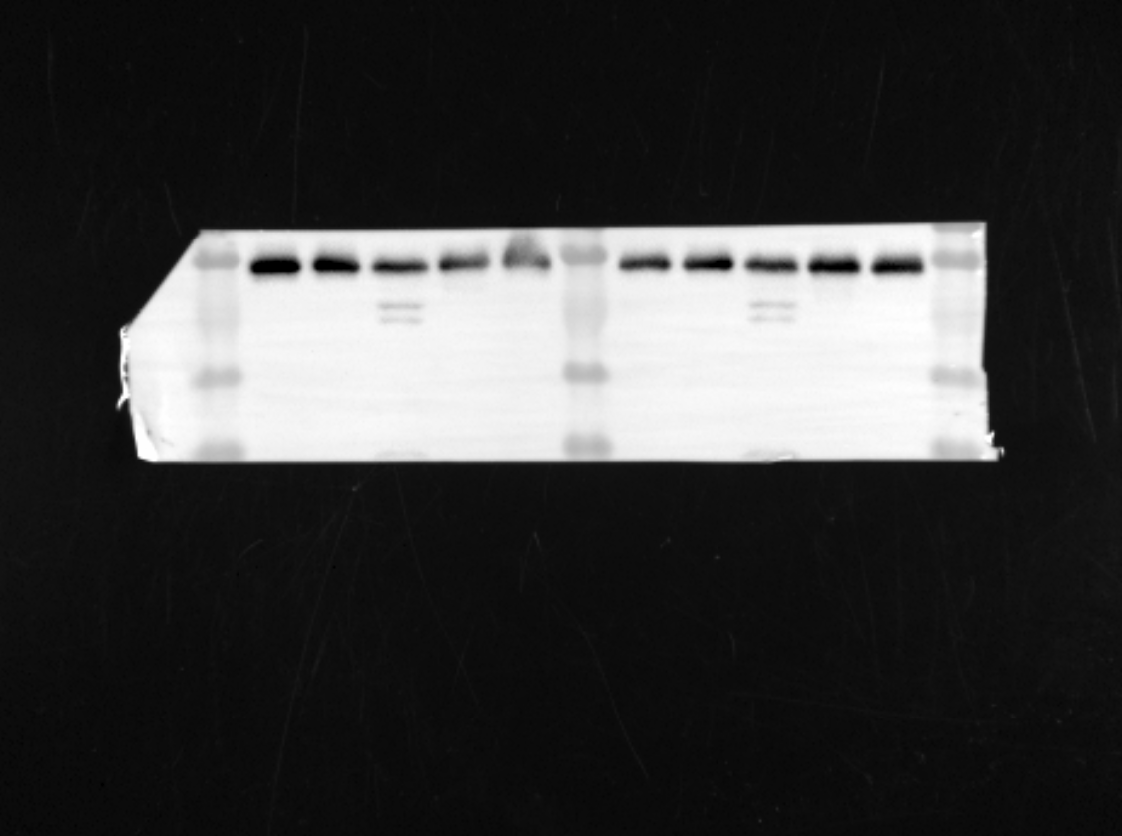

Supplement: Figure 2—source data 1. [file elife-84493-fig2-data1.zip › Figure 2-source data 1/The original files /gapdh.tif]

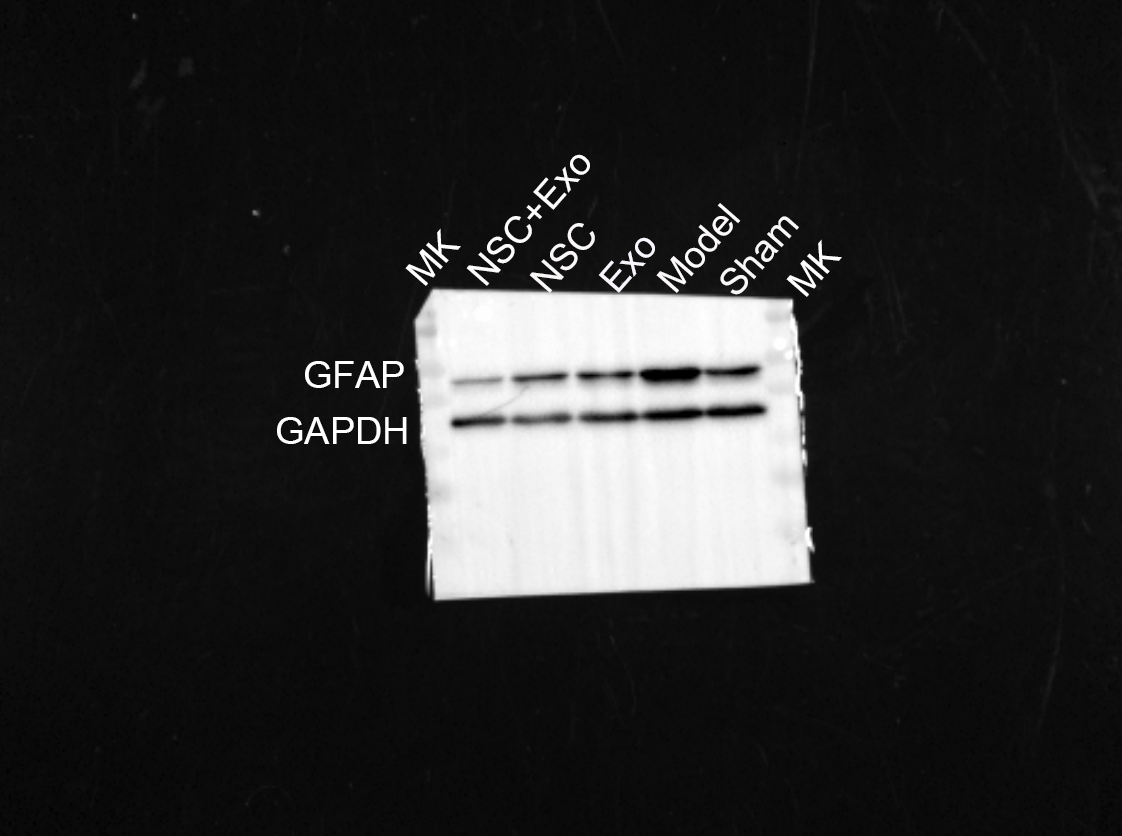

Supplement: Figure 2—source data 1. [file elife-84493-fig2-data1.zip › Figure 2-source data 1/The labeled files/GFAP+actin.tif]

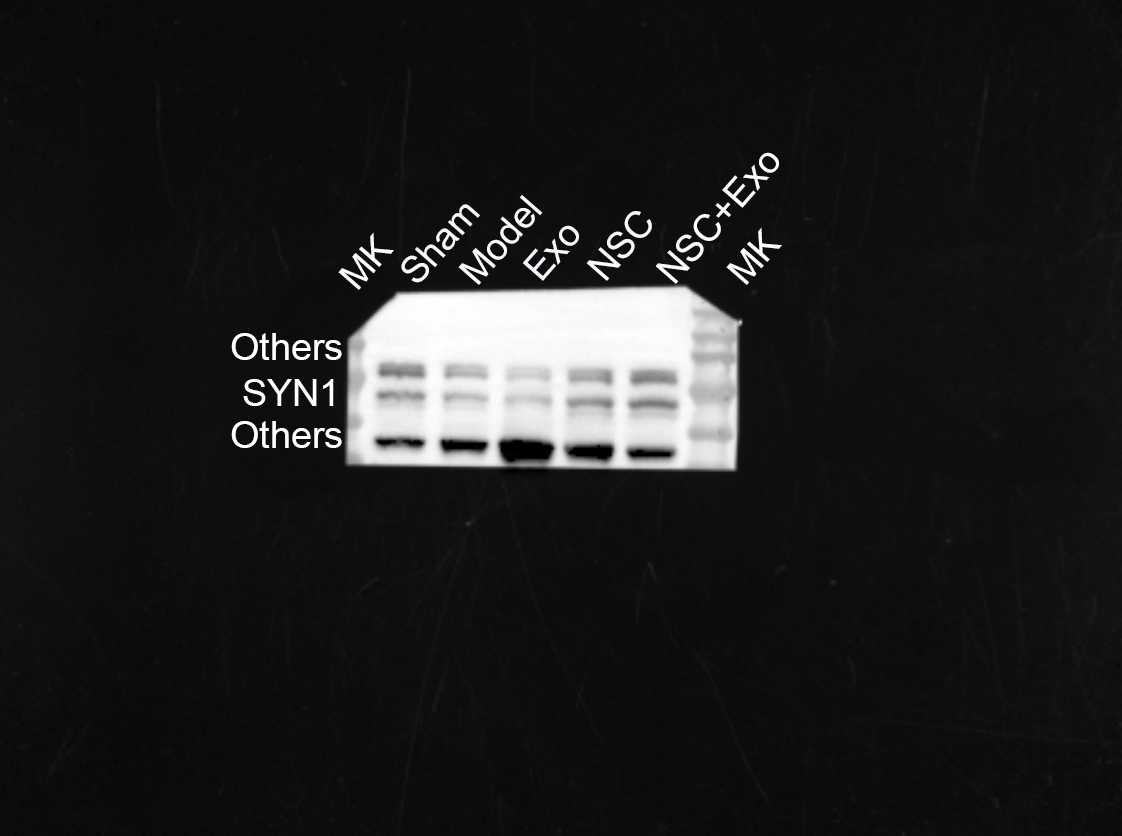

Supplement: Figure 2—source data 1. [file elife-84493-fig2-data1.zip › Figure 2-source data 1/The labeled files/syn.tif]

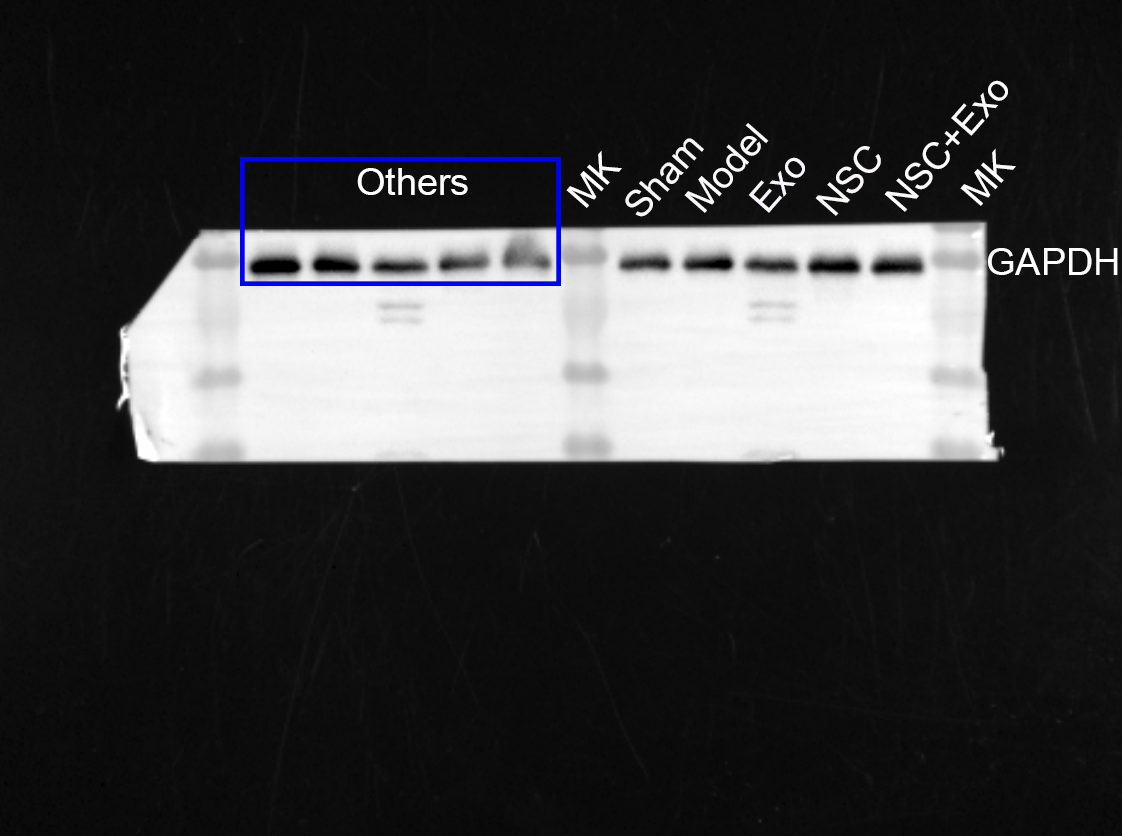

Supplement: Figure 2—source data 1. [file elife-84493-fig2-data1.zip › Figure 2-source data 1/The labeled files/gapdh.tif]

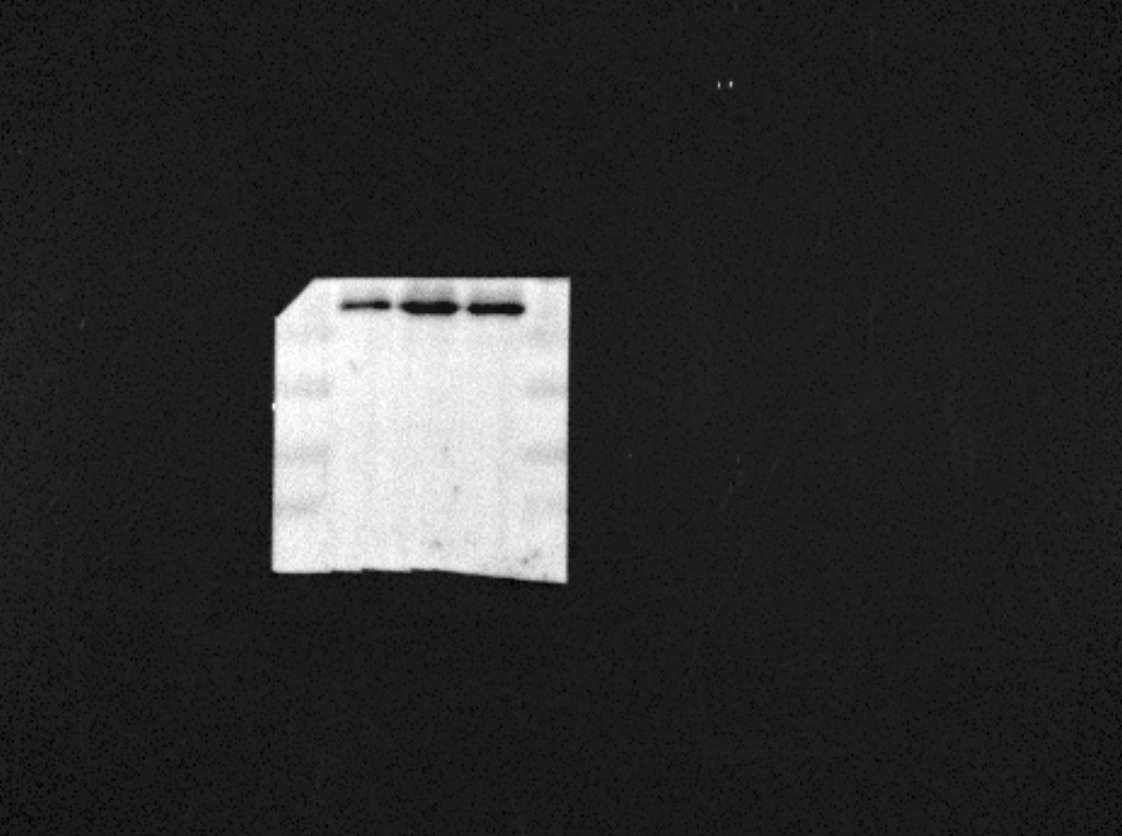

Supplement: Figure 2—figure supplement 1—source data 1. [file elife-84493-fig2-figsupp1-data1.zip › Figure 2 supplement 1 source data 1/The original files /caspase-3.tif]

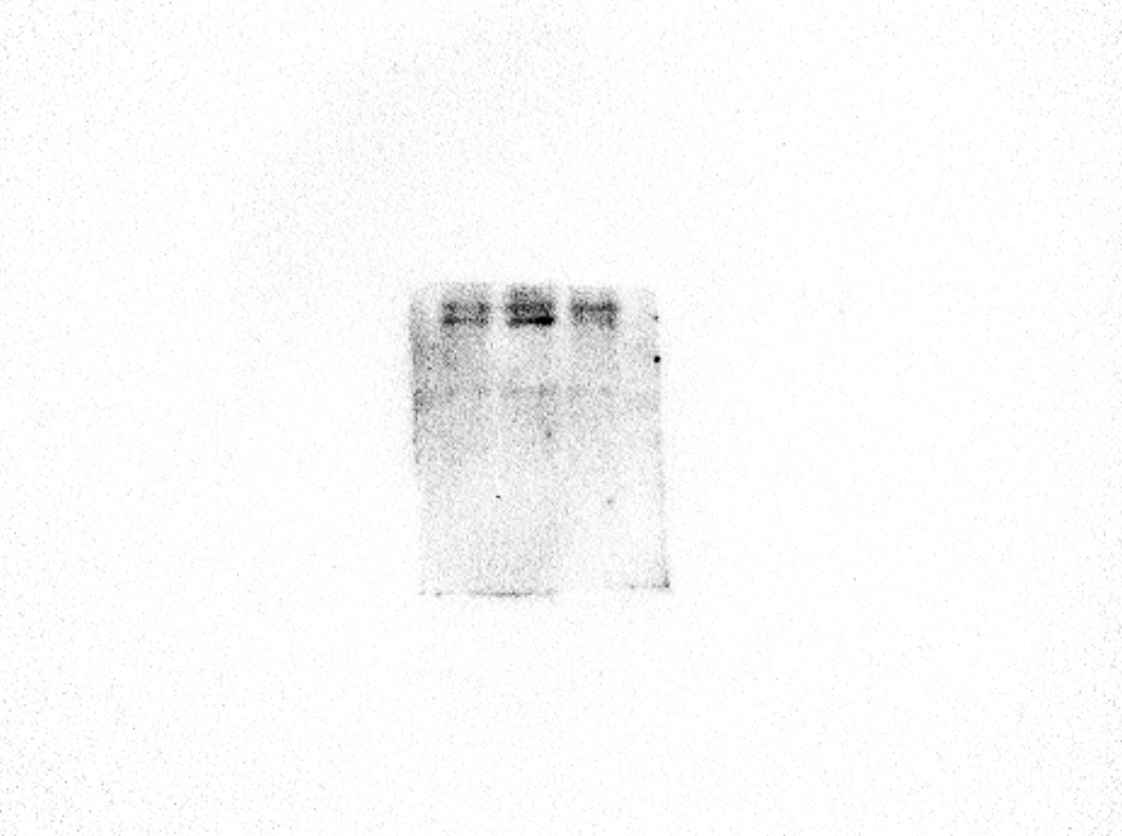

Supplement: Figure 2—figure supplement 1—source data 1. [file elife-84493-fig2-figsupp1-data1.zip › Figure 2 supplement 1 source data 1/The original files /c-caspase-3.tif]

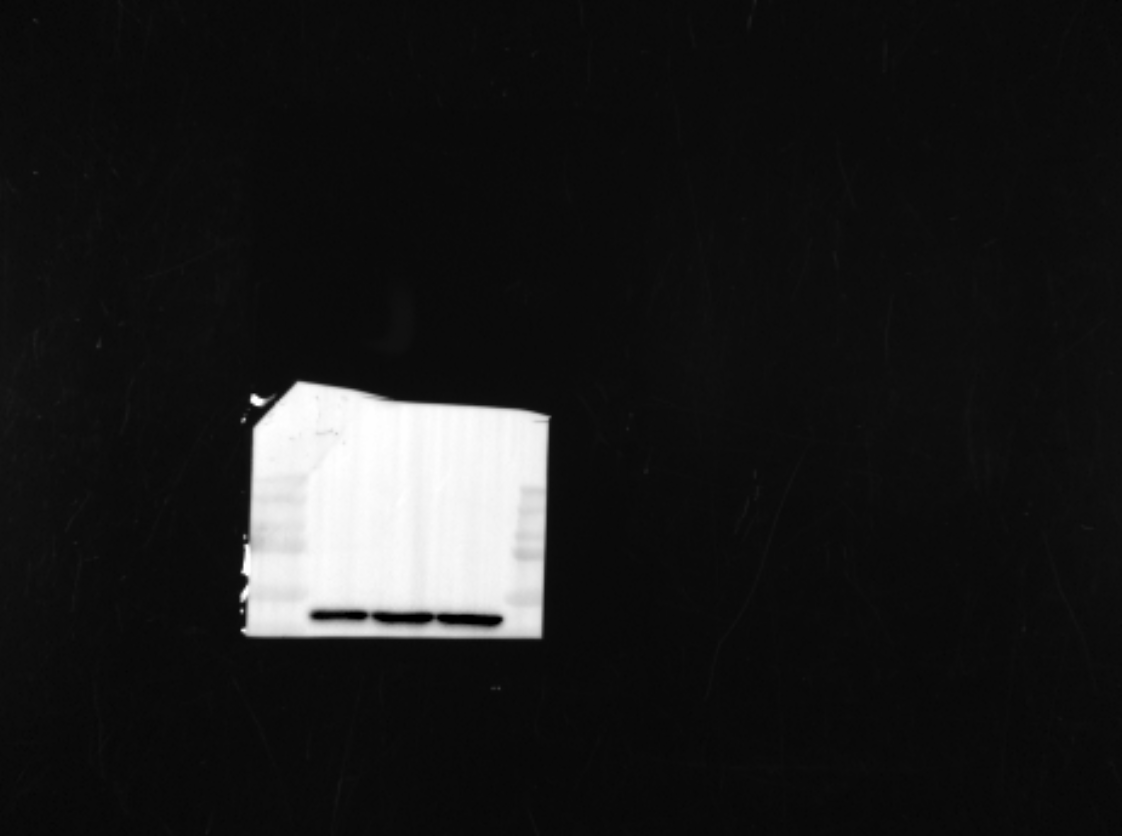

Supplement: Figure 2—figure supplement 1—source data 1. [file elife-84493-fig2-figsupp1-data1.zip › Figure 2 supplement 1 source data 1/The original files /╬▓-actin.tif]

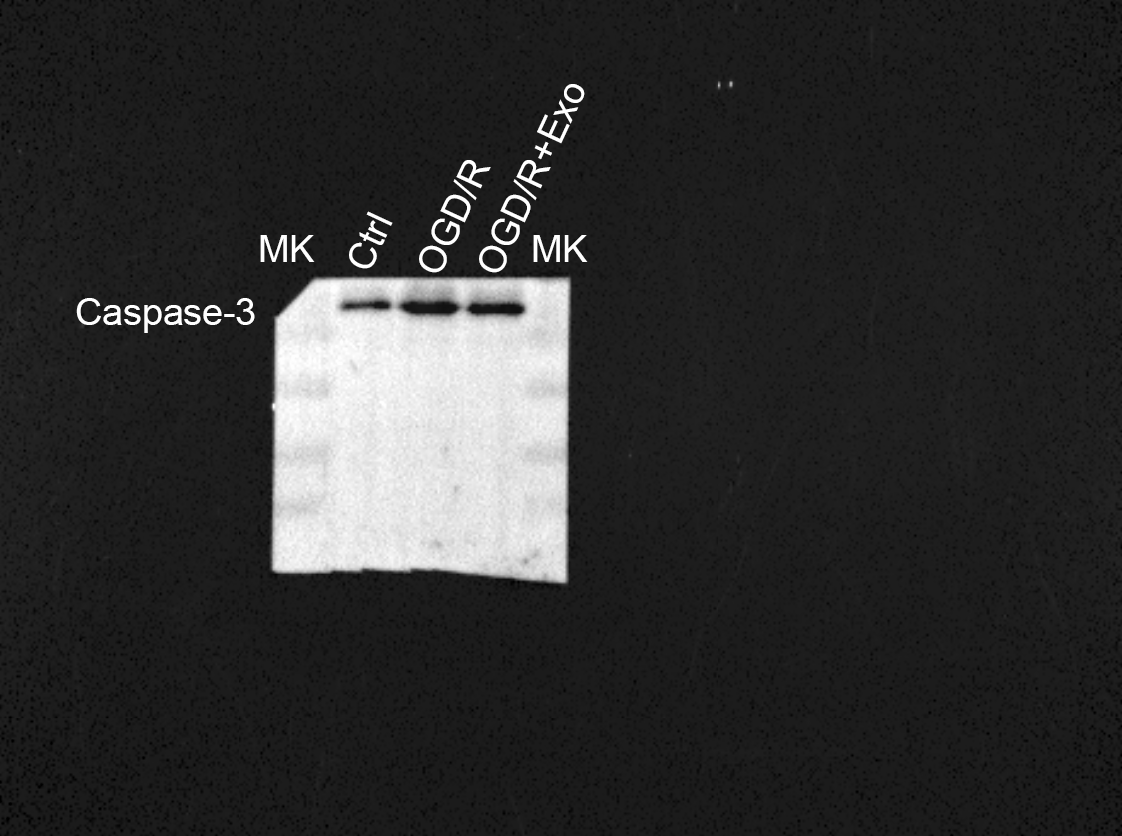

Supplement: Figure 2—figure supplement 1—source data 1. [file elife-84493-fig2-figsupp1-data1.zip › Figure 2 supplement 1 source data 1/The labeled files/caspase-3.tif]

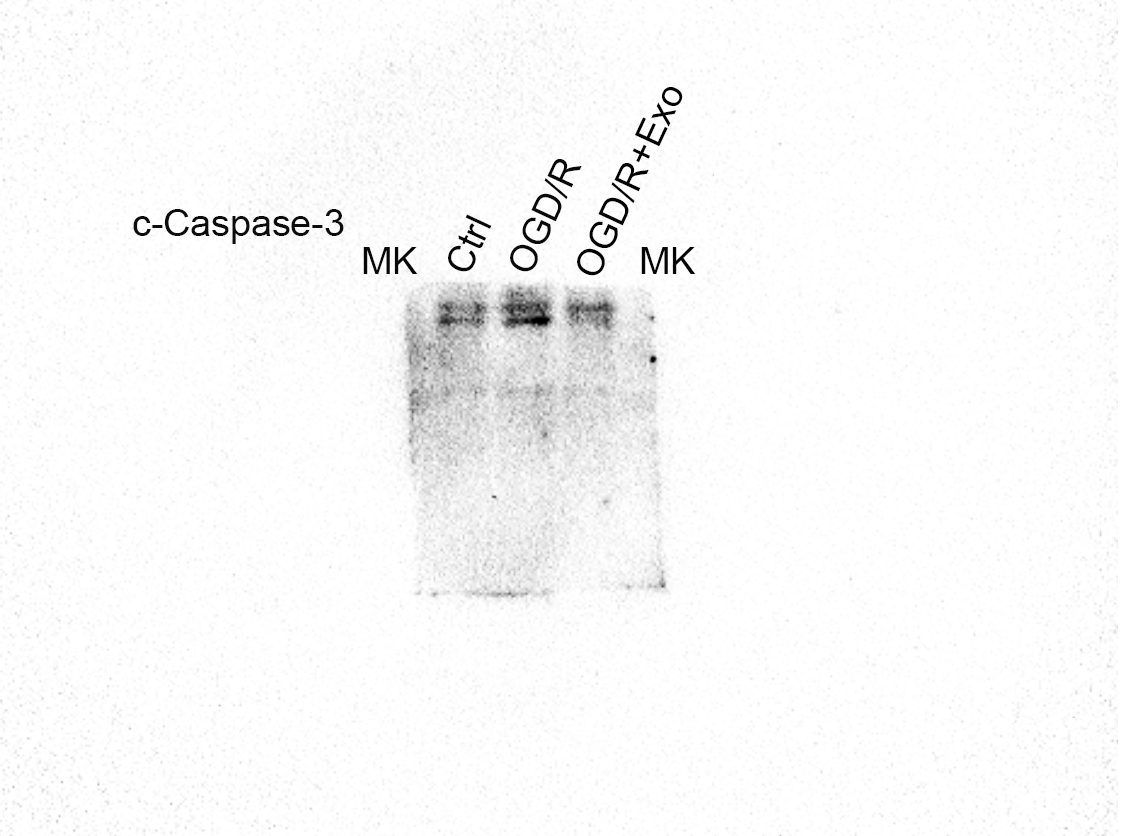

Supplement: Figure 2—figure supplement 1—source data 1. [file elife-84493-fig2-figsupp1-data1.zip › Figure 2 supplement 1 source data 1/The labeled files/c-caspase-3.tif]

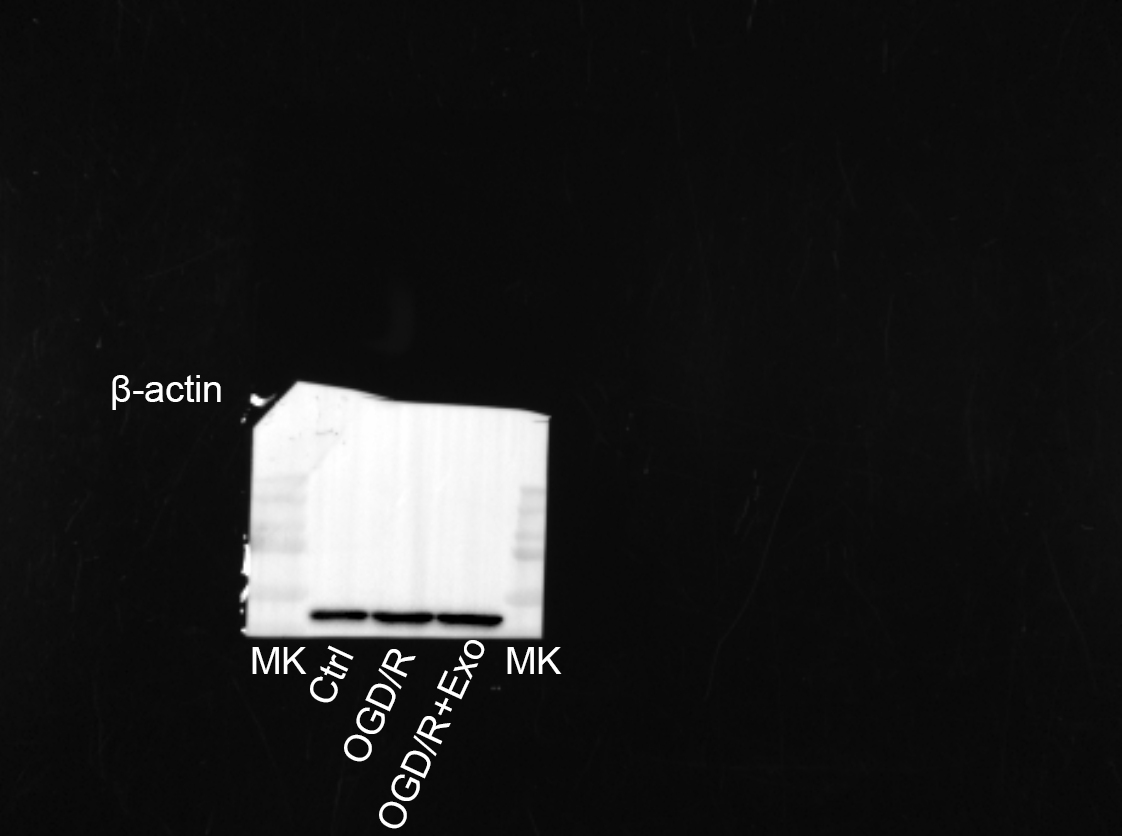

Supplement: Figure 2—figure supplement 1—source data 1. [file elife-84493-fig2-figsupp1-data1.zip › Figure 2 supplement 1 source data 1/The labeled files/╬▓-actin.tif]

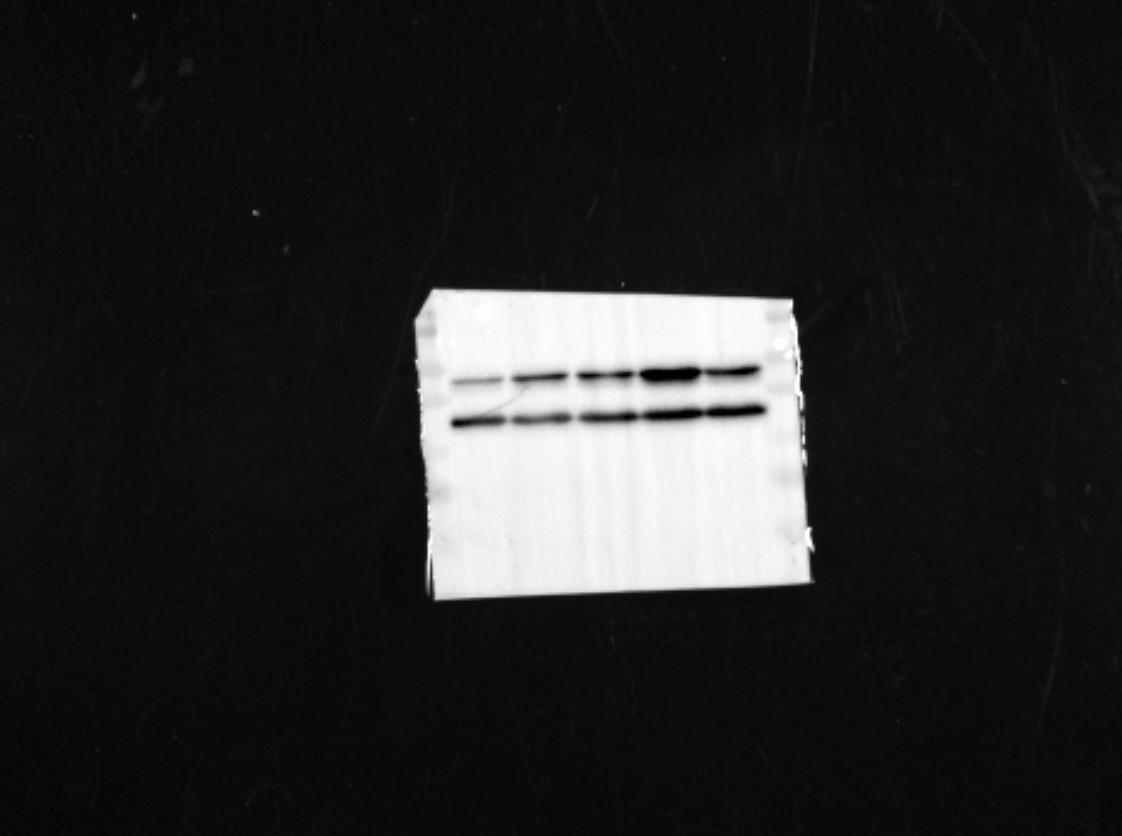

Supplement: Figure 4—source data 1. [file elife-84493-fig4-data1.zip › Figure 4-source data 1/The original files /GFAP+actin.tif]

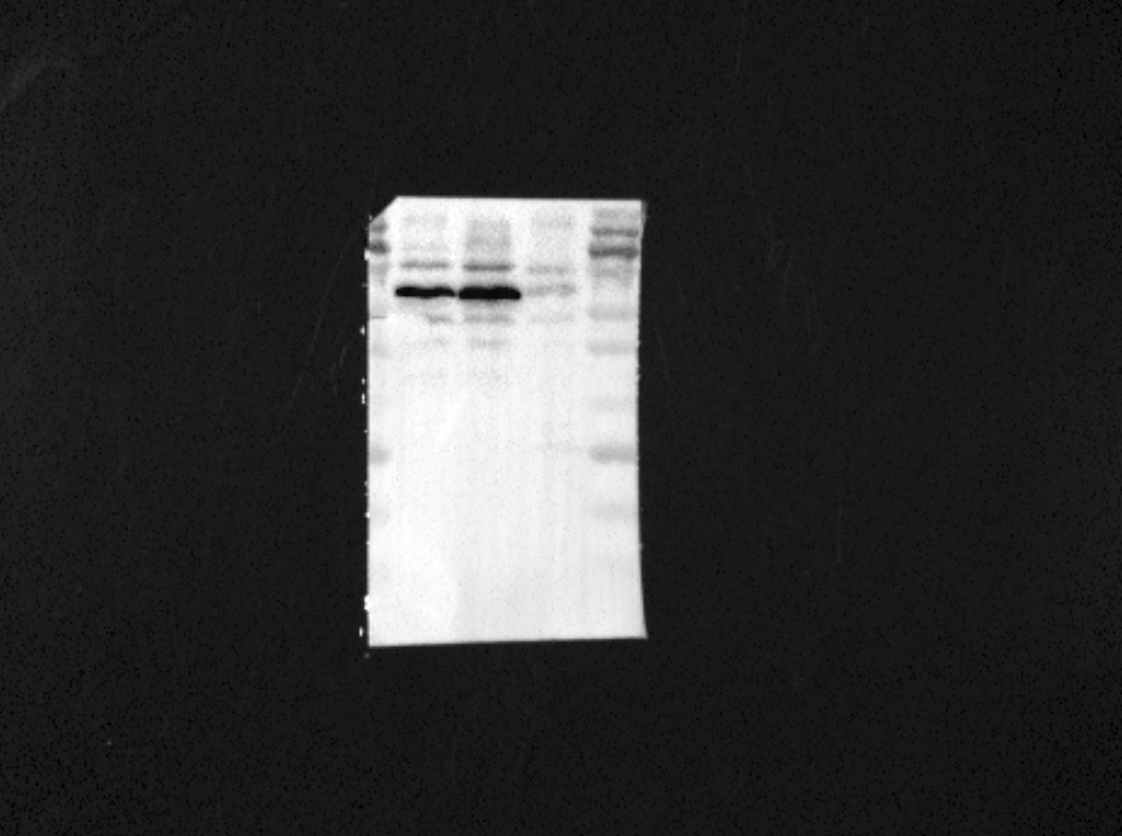

Supplement: Figure 5—figure supplement 1—source data 1. [file elife-84493-fig5-figsupp1-data1.zip › Figure 5 supplement 1-source data 1/The original files /PTPN1.tif]

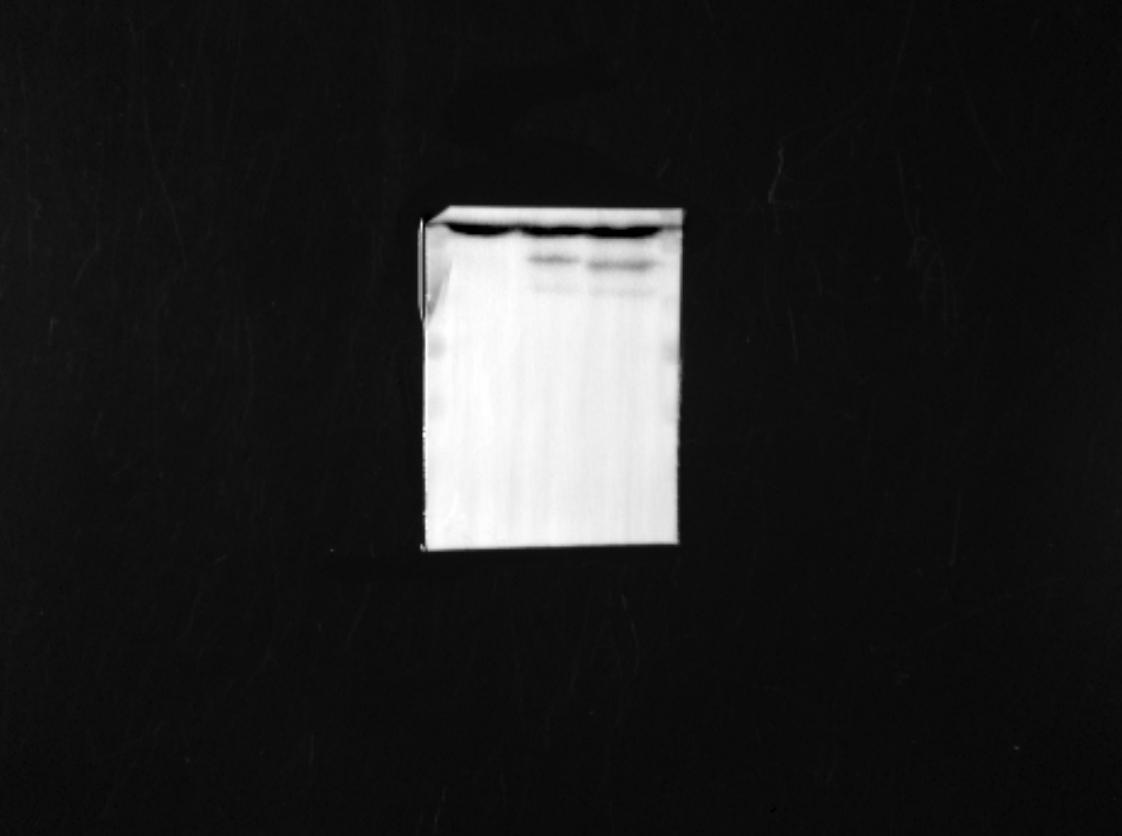

Supplement: Figure 5—figure supplement 1—source data 1. [file elife-84493-fig5-figsupp1-data1.zip › Figure 5 supplement 1-source data 1/The original files /╬▓-actin.tif]

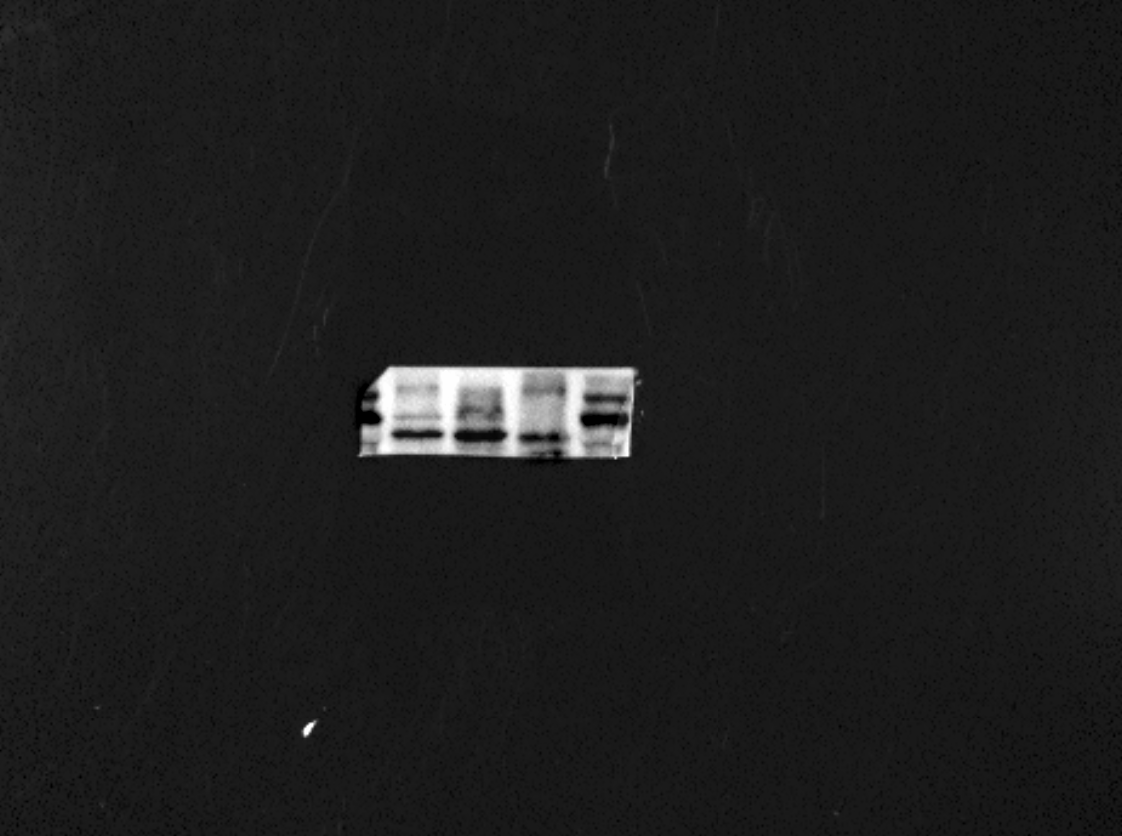

Supplement: Figure 5—figure supplement 1—source data 1. [file elife-84493-fig5-figsupp1-data1.zip › Figure 5 supplement 1-source data 1/The original files /IKK╬▒.tif]

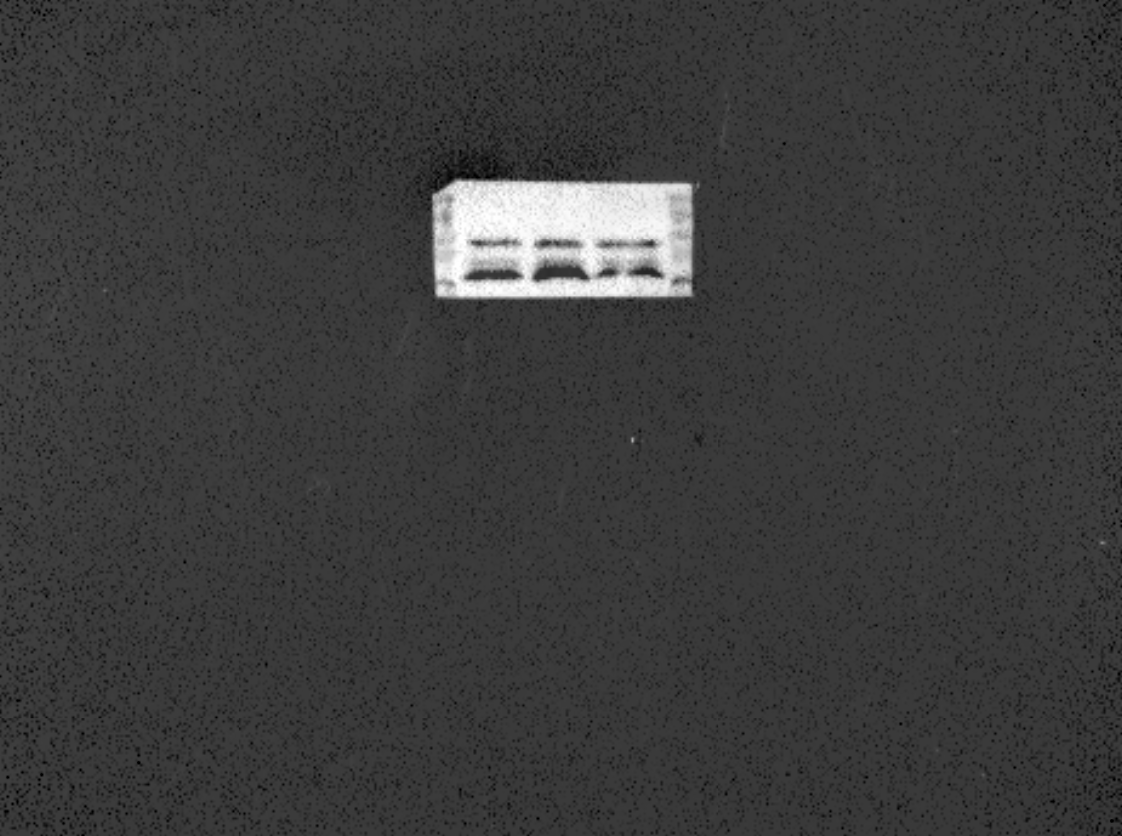

Supplement: Figure 5—figure supplement 1—source data 1. [file elife-84493-fig5-figsupp1-data1.zip › Figure 5 supplement 1-source data 1/The original files /STAT3.tif]

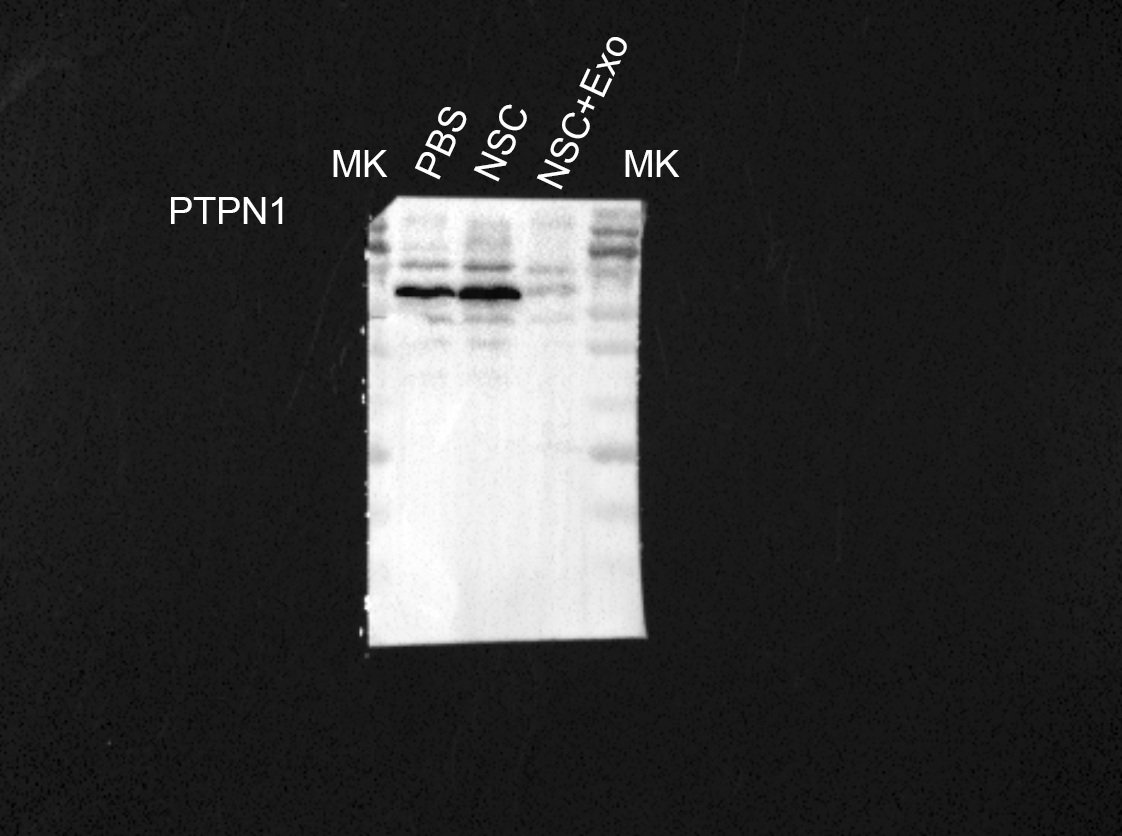

Supplement: Figure 5—figure supplement 1—source data 1. [file elife-84493-fig5-figsupp1-data1.zip › Figure 5 supplement 1-source data 1/The labeled files/PTPN1.tif]

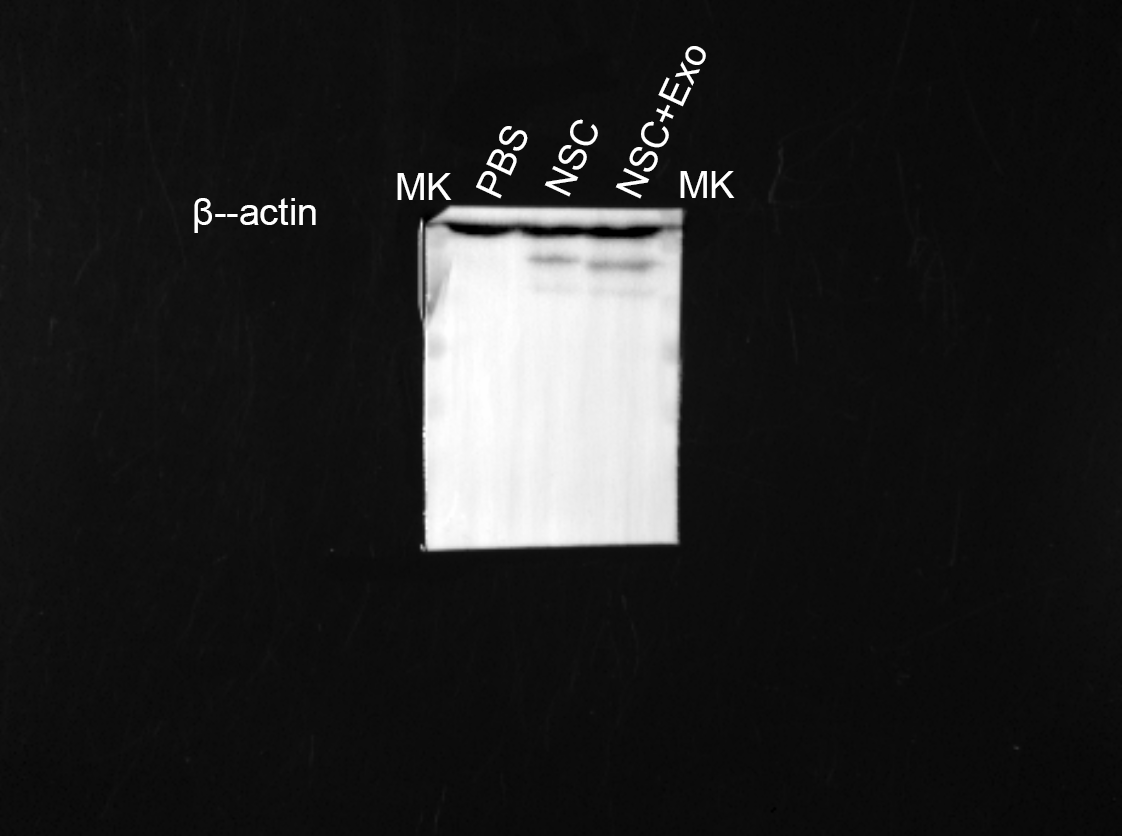

Supplement: Figure 5—figure supplement 1—source data 1. [file elife-84493-fig5-figsupp1-data1.zip › Figure 5 supplement 1-source data 1/The labeled files/╬▓-actin.tif]

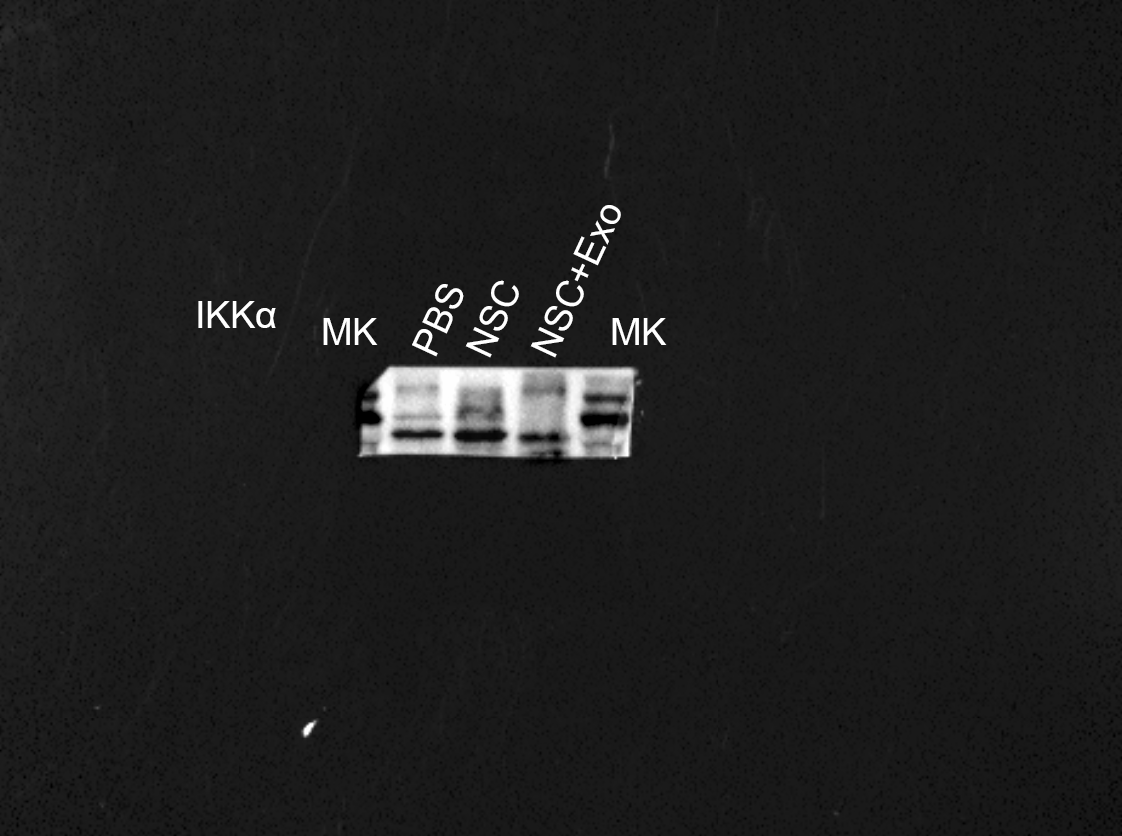

Supplement: Figure 5—figure supplement 1—source data 1. [file elife-84493-fig5-figsupp1-data1.zip › Figure 5 supplement 1-source data 1/The labeled files/IKK╬▒.tif]

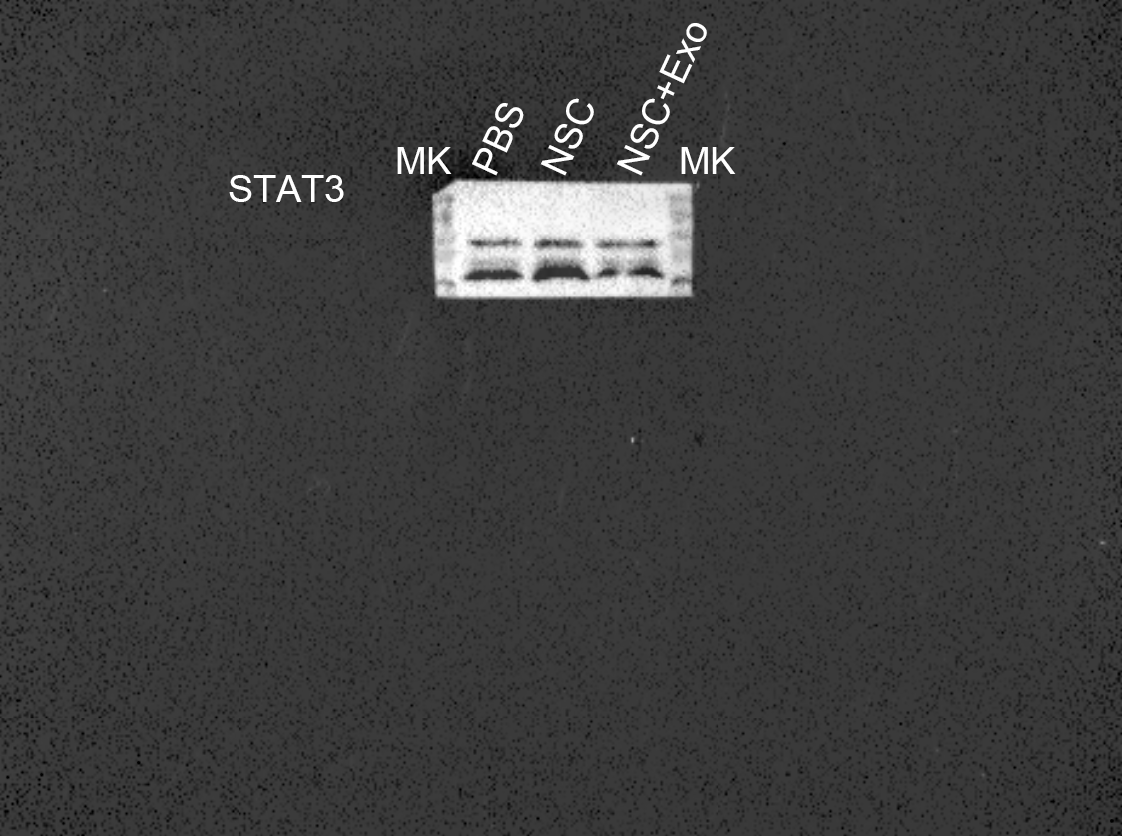

Supplement: Figure 5—figure supplement 1—source data 1. [file elife-84493-fig5-figsupp1-data1.zip › Figure 5 supplement 1-source data 1/The labeled files/STAT3.tif]
