# Supplementary material for: NSC-derived exosomes enhance therapeutic effects of NSC transplantation on cerebral ischemia in mice
Source: eLife. 2023 Apr 27;12:e84493. doi: 10.7554/eLife.84493 (PMC10139690; doi:10.7554/eLife.84493)
Supplement: Figure 1—figure supplement 2—source data 1. [file elife-84493-fig1-figsupp2-data1.zip › Figure 1 supplement 2-source data 1/Figure 1 supplement 2-source data 1.docx]

**Figure 1 supplement 2-Resource data: Behavioral data**

| **Balance beam** | **Sham** | | | | | | | | | | | | | | | | | | | | | | | | | | | | | | | | | | | | | | | | | | |  |  |  |  |
| --- | --- | --- | --- | --- | --- | --- | --- | --- | --- | --- | --- | --- | --- | --- | --- | --- | --- | --- | --- | --- | --- | --- | --- | --- | --- | --- | --- | --- | --- | --- | --- | --- | --- | --- | --- | --- | --- | --- | --- | --- | --- | --- | --- | --- | --- | --- | --- |
| 0w | 10.00 | | | 9.50 | | 9.50 | | | | 10.00 | | 9.50 | | | 9.50 | | | | 9.50 | | | | 10.00 | | | | 9.50 | | | | 9.67 | | | | 9.00 | | 9.67 | | | | | | |  |  |  |  |
| 1w | 10.00 | | | 10.00 | | 10.00 | | | | 9.33 | | 9.00 | | | 9.67 | | | | 9.33 | | | | 10.00 | | | | 9.67 | | | | 9.67 | | | | 10.00 | | 9.67 | | | | | | |  |  |  |  |
| 2w | 10.00 | | | 10.00 | | 10.00 | | | | 9.33 | | 9.00 | | | 10.00 | | | | 9.67 | | | | 9.67 | | | | 9.67 | | | | 9.67 | | | | 10.00 | | 9.67 | | | | | | |  |  |  |  |
| 3w | 9.00 | | | 9.67 | | 10.00 | | | | 9.67 | | 8.67 | | | 10.00 | | | | 10.00 | | | | 9.00 | | | | 9.67 | | | | 9.67 | | | | 10.00 | | 9.33 | | | | | | |  |  |  |  |
| 4w | 10.00 | | | 10.00 | | 10.00 | | | | 10.00 | | 9.33 | | | 10.00 | | | | 10.00 | | | | 8.67 | | | | 10.00 | | | | 9.67 | | | | 10.00 | | 9.00 | | | | | | |  |  |  |  |
| 5w | 9.50 | | | 9.83 | | 10.00 | | | | 9.83 | | 9.00 | | | 10.00 | | | | 10.00 | | | | 8.83 | | | | 9.83 | | | | 9.67 | | | | 10.00 | | 9.17 | | | | | | |  |  |  |  |
| 6w | 9.67 | | | 10.00 | | 10.00 | | | | 9.33 | | 9.67 | | | 9.33 | | | | 10.00 | | | | 9.67 | | | | 9.33 | | | | 9.33 | | | | 10.00 | | 9.67 | | | | | | |  |  |  |  |
| 7w | 9.58 | | | 9.92 | | 10.00 | | | | 9.58 | | 9.33 | | | 9.67 | | | | 10.00 | | | | 9.25 | | | | 9.58 | | | | 9.50 | | | | 10.00 | | 9.42 | | | | | | |  |  |  |  |
| 8w | 10.00 | | | 10.00 | | 10.00 | | | | 9.67 | | 9.00 | | | 9.67 | | | | 10.00 | | | | 9.67 | | | | 9.67 | | | | 9.67 | | | | 10.00 | | 9.33 | | | | | | |  |  |  |  |
|  |  | | |  | | |  | | | |  | |  | | | |  | | | |  | | | |  | | | |  | | | |  | |  | | | | |  | | | |  |  |  |  |
| **Balance beam** | **Model** | | | | | | | | | | | | | | | | | | | | | | | | | | | | | | | | | | | | | | | | | | |  |  |  |  |
| 0w | 8.67 | | | 1.67 | | 9.00 | | | | 5.00 | | 5.33 | | | 9.00 | | | | 9.17 | | | | 9.00 | | | | 5.33 | | | | 4.33 | | | | 9.00 | | 8.67 | | | | | | |  | | | |
| 1w | 8.67 | | | 3.00 | | 7.33 | | | | 6.33 | | 4.67 | | | 7.00 | | | | 8.33 | | | | 8.67 | | | | 5.67 | | | | 5.00 | | | | 9.00 | | 4.33 | | | | | | |  | | | |
| 2w | 7.00 | | | 2.67 | | 9.33 | | | | 5.33 | | 5.67 | | | 8.00 | | | | 7.00 | | | | 8.33 | | | | 6.67 | | | | 5.67 | | | | 9.00 | | 6.00 | | | | | | |  | | | |
| 3w | 7.00 | | | 4.00 | | 9.00 | | | | 8.00 | | 4.33 | | | 8.67 | | | | 7.33 | | | | 8.67 | | | | 6.33 | | | | 7.33 | | | | 7.00 | | 5.67 | | | | | | |  | | | |
| 4w | 6.00 | | | 2.67 | | 9.67 | | | | 7.00 | | 6.00 | | | 8.00 | | | | 7.33 | | | | 9.00 | | | | 6.00 | | | | 7.33 | | | | 9.33 | | 8.33 | | | | | | |  | | | |
| 5w | 6.67 | | | 4.00 | | 9.33 | | | | 7.67 | | 7.00 | | | 8.33 | | | | 9.00 | | | | 8.00 | | | | 7.33 | | | | 7.00 | | | | 7.33 | | 8.33 | | | | | | |  | | | |
| 6w | 6.67 | | | 5.33 | | 7.67 | | | | 6.67 | | 7.00 | | | 6.67 | | | | 9.67 | | | | 7.67 | | | | 8.00 | | | | 7.67 | | | | 9.00 | | 6.67 | | | | | | |  | | | |
| 7w | 6.83 | | | 6.00 | | 8.00 | | | | 6.50 | | 7.00 | | | 7.00 | | | | 8.67 | | | | 7.50 | | | | 7.83 | | | | 7.83 | | | | 8.33 | | 7.17 | | | | | | |  | | | |
| 8w | 7.00 | | | 6.67 | | 8.33 | | | | 6.33 | | 7.00 | | | 7.33 | | | | 7.67 | | | | 7.33 | | | | 7.67 | | | | 8.00 | | | | 7.67 | | 7.67 | | | | | | |  | | | |
|  |  | | |  | | |  | | | |  | |  | | | |  | | | |  | | | |  | | | |  | | | |  | |  | | | | |  | | | |  | | | |
| **Balance beam** | **Exo** | | | | | | | | | | | | | | | | | | | | | | | | | | | | | | | | | | | | | | | | | | |  | | | |
| 0w | 7.00 | | | 7.00 | | 6.33 | | | | 8.33 | | 7.00 | | | 4.33 | | | | 6.33 | | | | 9.67 | | | | 7.67 | | | | 8.33 | | | | 8.67 | | 7.67 | | | | | | |  | | | |
| 1w | 7.33 | | | 6.33 | | 8.67 | | | | 7.33 | | 8.00 | | | 6.67 | | | | 4.33 | | | | 8.33 | | | | 8.67 | | | | 4.00 | | | | 7.00 | | 8.00 | | | | | | |  | | | |
| 2w | 7.00 | | | 7.67 | | 8.67 | | | | 7.00 | | 5.67 | | | 5.33 | | | | 8.33 | | | | 9.33 | | | | 5.67 | | | | 8.00 | | | | 5.33 | | 6.67 | | | | | | |  | | | |
| 3w | 8.33 | | | 5.00 | | 6.67 | | | | 6.67 | | 9.33 | | | 9.00 | | | | 9.33 | | | | 8.67 | | | | 9.33 | | | | 6.00 | | | | 5.00 | | 4.33 | | | | | | |  | | | |
| 4w | 8.00 | | | 6.00 | | 5.00 | | | | 8.67 | | 9.67 | | | 7.67 | | | | 5.33 | | | | 9.00 | | | | 9.67 | | | | 5.67 | | | | 6.33 | | 6.67 | | | | | | |  | | | |
| 5w | 8.00 | | | 7.67 | | 7.33 | | | | 7.33 | | 5.67 | | | 6.67 | | | | 5.33 | | | | 8.67 | | | | 9.67 | | | | 9.67 | | | | 7.33 | | 7.67 | | | | | | |  | | | |
| 6w | 7.67 | | | 8.67 | | 6.67 | | | | 9.67 | | 8.67 | | | 6.67 | | | | 5.33 | | | | 8.67 | | | | 8.67 | | | | 5.00 | | | | 7.67 | | 7.00 | | | | | | |  | | | |
| 7w | 8.17 | | | 6.67 | | 7.67 | | | | 9.00 | | 8.83 | | | 7.33 | | | | 4.50 | | | | 8.67 | | | | 8.67 | | | | 6.67 | | | | 6.00 | | 6.83 | | | | | | |  | | | |
| 8w | 8.67 | | | 8.00 | | 8.67 | | | | 8.33 | | 9.00 | | | 8.00 | | | | 6.67 | | | | 8.67 | | | | 8.67 | | | | 8.33 | | | | 8.33 | | 6.67 | | | | | | |  | | | |
|  |  | | |  | | |  | | | |  | |  | | | |  | | | |  | | | |  | | | |  | | | |  | |  | | | | |  | | | |  | | | |
| **Balance beam** | **NSC** | | | | | | | | | | | | | | | | | | | | | | | | | | | | | | | | | |  | | |  | | | | | |  | | | |
| 0w | 6.33 | | | 9.33 | | 8.67 | | | | 7.33 | | 6.33 | | | 5.33 | | | | 5.33 | | | | 5.33 | | | | 8.33 | | | | 8.67 | | | |  | |  | | | | | |  | | |  |  |
| *Continued* | | | | | | | | | | | | | | | | | | | | | | | | | | | | | | | | | | |  | |  | | | | | |  | | |  |  |
| 1w | 6.33 | | | 8.67 | | 9.67 | | | | 9.33 | | 9.33 | | | 5.33 | | | | 5.33 | | | | 7.67 | | | | 8.00 | | | | 9.33 | | | |  | |  | | | | | |  | | |  |  |
| 2w | 8.67 | | | 8.00 | | 10.00 | | | | 9.33 | | 8.67 | | | 6.33 | | | | 7.33 | | | | 9.00 | | | | 9.33 | | | | 7.67 | | | |  | |  | | | | | |  | | |  |  |
| 3w | 9.33 | | | 8 | | 9.33 | | | | 8.67 | | 8.67 | | | 7.00 | | | | 7.00 | | | | 9.00 | | | | 8.67 | | | | 7.67 | | | |  | |  | | | | | |  | | |  |  |
| 4w | 8.67 | | | 7.67 | | 9.33 | | | | 9.33 | | 9.33 | | | 9.33 | | | | 8.33 | | | | 8.33 | | | | 8.33 | | | | 8.00 | | | |  | |  | | | | | |  | | |  |  |
| 5w | 9.33 | | | 9.33 | | 9.33 | | | | 8.67 | | 9.33 | | | 9.00 | | | | 8.33 | | | | 8.33 | | | | 8.67 | | | | 8.67 | | | |  | |  | | | | | |  | | |  |  |
| 6w | 9.33 | | | 10.00 | | 9.33 | | | | 9.33 | | 9.67 | | | 9.00 | | | | 9.00 | | | | 10.00 | | | | 9.33 | | | | 8.67 | | | |  | |  | | | | | |  | | |  |  |
| 7w | 9.83 | | | 8.33 | | 9.33 | | | | 9.50 | | 10.00 | | | 8.83 | | | | 9.17 | | | | 9.00 | | | | 9.17 | | | | 8.50 | | | |  | |  | | | | | |  | | |  |  |
| 8w | 9.67 | | | 7.67 | | 9.33 | | | | 9.67 | | 10.00 | | | 8.33 | | | | 8.67 | | | | 9.00 | | | | 9.67 | | | | 7.67 | | | |  | |  | | | | | |  | | |  |  |
|  |  | | |  | | |  | | | |  | |  | | | |  | | | |  | | | |  | | | |  | | | |  | |  | | | | |  | | | |  | | | |
| **Balance beam** | **NSC+Exo** | | | | | | | | | | | | | | | | | | | | | | | | | | | | | | | | | | | | | | | | | | |  | | | |
| 0w | 8.33 | | | 8.67 | | 7.33 | | | | 7.33 | | 9.33 | | | 7.67 | | | | 6.00 | | | | 9.33 | | | | 8.33 | | | | 5.67 | | | | 8.33 | | 3.00 | | | | | | |  | | | |
| 1w | 9.33 | | | 9.33 | | 9.33 | | | | 7.33 | | 9.67 | | | 7.33 | | | | 9.67 | | | | 9.00 | | | | 7.67 | | | | 5.33 | | | | 9.00 | | 10.00 | | | | | | |  | | | |
| 2w | 9.33 | | | 9.00 | | 8.33 | | | | 8.33 | | 9.33 | | | 8.00 | | | | 10.00 | | | | 8.67 | | | | 9.00 | | | | 6.33 | | | | 9.00 | | 9.00 | | | | | | |  | | | |
| 3w | 9.67 | | | 9.67 | | 9.67 | | | | 7.67 | | 10.00 | | | 9.00 | | | | 10.00 | | | | 9.67 | | | | 8.33 | | | | 7.67 | | | | 10.00 | | 9.00 | | | | | | |  | | | |
| 4w | 9.00 | | | 9.00 | | 9.33 | | | | 10.00 | | 9.33 | | | 8.00 | | | | 9.67 | | | | 10.00 | | | | 8.67 | | | | 10.00 | | | | 10.00 | | 9.67 | | | | | | |  | | | |
| 5w | 9.00 | | | 9.33 | | 9.00 | | | | 9.00 | | 9.00 | | | 10.00 | | | | 9.67 | | | | 10.00 | | | | 8.00 | | | | 8.67 | | | | 9.33 | | 9.33 | | | | | | |  | | | |
| 6w | 10.00 | | | 9.67 | | 9.33 | | | | 9.33 | | 9.33 | | | 9.00 | | | | 9.67 | | | | 9.33 | | | | 9.00 | | | | 9.00 | | | | 9.67 | | 9.33 | | | | | | |  | | | |
| 7w | 9.83 | | | 9.83 | | 9.33 | | | | 9.50 | | 9.50 | | | 9.00 | | | | 9.67 | | | | 9.50 | | | | 9.00 | | | | 8.67 | | | | 9.83 | | 9.67 | | | | | | |  | | | |
| 8w | 9.67 | | | 10.00 | | 9.33 | | | | 9.67 | | 9.67 | | | 9.00 | | | | 9.67 | | | | 9.67 | | | | 9.00 | | | | 8.33 | | | | 10.00 | | 10.00 | | | | | | |  | | | |
|  |  | | | | | | | | | | | | | | | | | | | | | | | | | | | | | | | | | | | | | | | | | | |  |  |  |  |
| **Ladder lung** | **Sham** | | | | | | | | | | | | | | | | | | | | | | | | | | | | | | | | | | | | | | | | | | |  |  |  |  |
| 0w | 10.00 | | | 9.50 | | 10.00 | | | | 9.50 | | 10.00 | | | 9.50 | | | | 10.00 | | | | 9.50 | | | | 9.50 | | | | 9.50 | | | | 9.50 | | 9.00 | | | | | | |  |  |  |  |
| 1w | 10.00 | | | 9.50 | | 9.50 | | | | 10.00 | | 10.00 | | | 9.50 | | | | 9.50 | | | | 10.00 | | | | 10.00 | | | | 10.00 | | | | 10.00 | | 10.00 | | | | | | |  |  |  |  |
| 2w | 9.50 | | | 10.00 | | 9.50 | | | | 10.00 | | 10.00 | | | 10.00 | | | | 10.00 | | | | 10.00 | | | | 10.00 | | | | 10.00 | | | | 9.50 | | 10.00 | | | | | | |  |  |  |  |
| 3w | 9.00 | | | 10.00 | | 9.50 | | | | 9.50 | | 10.00 | | | 10.00 | | | | 10.00 | | | | 10.00 | | | | 9.50 | | | | 10.00 | | | | 10.00 | | 10.00 | | | | | | |  |  |  |  |
| 4w | 9.25 | | | 10.00 | | 9.50 | | | | 9.75 | | 10.00 | | | 10.00 | | | | 10.00 | | | | 10.00 | | | | 9.75 | | | | 10.00 | | | | 9.75 | | 10.00 | | | | | | |  |  |  |  |
| 5w | 9.00 | | | 10.00 | | 10.00 | | | | 9.50 | | 10.00 | | | 9.50 | | | | 10.00 | | | | 10.00 | | | | 9.50 | | | | 10.00 | | | | 10.00 | | 10.00 | | | | | | |  |  |  |  |
| 6w | 9.13 | | | 10.00 | | 9.75 | | | | 9.63 | | 10.00 | | | 9.75 | | | | 10.00 | | | | 10.00 | | | | 9.63 | | | | 10.00 | | | | 9.88 | | 10.00 | | | | | | |  |  |  |  |
| 7w | 10.00 | | | 9.00 | | 9.50 | | | | 10.00 | | 10.00 | | | 9.50 | | | | 9.50 | | | | 10.00 | | | | 10.00 | | | | 10.00 | | | | 10.00 | | 10.00 | | | | | | |  |  |  |  |
| 8w | 10.00 | | | 10.00 | | 10.00 | | | | 10.00 | | 10.00 | | | 9.50 | | | | 10.00 | | | | 10.00 | | | | 10.00 | | | | 10.00 | | | | 10.00 | | 10.00 | | | | | | |  |  |  |  |
|  |  |  | | | | | |  | |  | | | |  | | | |  | | | |  | | | |  | | | |  | | | |  |  | | | | | |  | | |  |  |  |  |
| **Ladder lung** | **Model** | | | | | | | | | | | | | | | | | | | | | | | | | | | | | | | | | | | | | | | | | | |  |  |  |  |
| 0w | 7.00 | | | 9.00 | | 8.50 | | | | 8.50 | | 5.00 | | | 7.00 | | | | 3.50 | | | | 6.33 | | | | 6.67 | | | | 8.00 | | | | 7.00 | | 7.50 | | | | | | |  | | | |
| 1w | 8.00 | | | 9.00 | | 7.50 | | | | 9.00 | | 6.50 | | | 4.00 | | | | 7.00 | | | | 6.50 | | | | 9.50 | | | | 9.00 | | | | 8.33 | | 5.33 | | | | | | |  | | | |
| 2w | 8.00 | | | 6.00 | | 9.50 | | | | 9.00 | | 8.00 | | | 5.00 | | | | 6.50 | | | | 8.50 | | | | 6.00 | | | | 8.00 | | | | 8.50 | | 6.50 | | | | | | |  | | | |
| *Continued* | | | | | | | | | | | | | | | | | | | | | | | | | | | | | | | | | | | | | | | | | | | |  | | | |
| 3w | 7.50 | | | 6.50 | | 8.00 | | | | 9.00 | | 6.67 | | | 6.50 | | | | 8.00 | | | | 8.00 | | | | 8.50 | | | | 5.50 | | | | 8.50 | | 5.50 | | | | | | |  | | | |
| 4w | 9.00 | | | 9.50 | | 9.00 | | | | 9.50 | | 7.00 | | | 2.50 | | | | 6.00 | | | | 7.50 | | | | 6.00 | | | | 8.33 | | | | 7.00 | | 7.00 | | | | | | |  | | | |
| 5w | 7.50 | | | 6.50 | | 9.00 | | | | 9.00 | | 7.00 | | | 6.50 | | | | 6.00 | | | | 7.50 | | | | 9.00 | | | | 9.50 | | | | 7.00 | | 6.50 | | | | | | |  | | | |
| 6w | 7.50 | | | 8.50 | | 8.50 | | | | 8.00 | | 6.00 | | | 4.50 | | | | 8.00 | | | | 8.00 | | | | 7.00 | | | | 7.50 | | | | 8.00 | | 6.00 | | | | | | |  | | | |
| 7w | 8.00 | | | 8.75 | | 8.75 | | | | 8.25 | | 6.25 | | | 6.25 | | | | 8.00 | | | | 8.25 | | | | 7.75 | | | | 8.25 | | | | 8.00 | | 7.25 | | | | | | |  | | | |
| 8w | 8.5 | | | 7 | | 7 | | | | 8.5 | | 6.5 | | | 8 | | | | 8 | | | | 8.5 | | | | 8.5 | | | | 9 | | | | 8 | | 8.5 | | | | | | |  | | | |
|  |  |  | | | | | |  | |  | | | |  | | | |  | | | |  | | | |  | | | |  | | | |  |  | | | | | |  | | |  | | | |
| **Ladder lung** | **Exo** | | | | | | | | | | | | | | | | | | | | | | | | | | | | | | | | | | | | | | | | | | |  | | | |
| 0w | 8.50 | | | 6.00 | | 3.00 | | | | 5.00 | | 9.00 | | | 3.00 | | | | 5.50 | | | | 9.00 | | | | 9.00 | | | | 8.00 | | | | 8.50 | | 8.00 | | | | | | |  | | | |
| 1w | 7.00 | | | 8.50 | | 6.50 | | | | 6.33 | | 9.00 | | | 7.50 | | | | 3.50 | | | | 3.50 | | | | 8.50 | | | | 10.00 | | | | 7.50 | | 8.50 | | | | | | |  | | | |
| 2w | 8.00 | | | 8.50 | | 6.50 | | | | 6.00 | | 8.50 | | | 6.50 | | | | 6.00 | | | | 8.50 | | | | 9.00 | | | | 8.50 | | | | 7.00 | | 8.50 | | | | | | |  | | | |
| 3w | 9.00 | | | 9.00 | | 8.00 | | | | 5.00 | | 9.00 | | | 6.50 | | | | 8.00 | | | | 9.50 | | | | 8.00 | | | | 4.00 | | | | 7.50 | | 9.00 | | | | | | |  | | | |
| 4w | 7.67 | | | 9.50 | | 7.00 | | | | 5.33 | | 9.00 | | | 5.33 | | | | 8.50 | | | | 9.50 | | | | 8.33 | | | | 5.00 | | | | 8.00 | | 6.33 | | | | | | |  | | | |
| 5w | 7.00 | | | 8.50 | | 10.00 | | | | 8.00 | | 9.00 | | | 7.50 | | | | 4.00 | | | | 9.50 | | | | 8.00 | | | | 6.00 | | | | 5.00 | | 8.00 | | | | | | |  | | | |
| 6w | 7.00 | | | 8.00 | | 9.50 | | | | 10.00 | | 10.00 | | | 7.00 | | | | 5.67 | | | | 9.00 | | | | 9.50 | | | | 6.00 | | | | 8.00 | | 8.00 | | | | | | |  | | | |
| 7w | 7.17 | | | 8.00 | | 9.75 | | | | 9.00 | | 9.50 | | | 7.00 | | | | 6.83 | | | | 9.25 | | | | 9.00 | | | | 7.00 | | | | 8.00 | | 8.00 | | | | | | |  | | | |
| 8w | 7.33 | | | 8.00 | | 10.00 | | | | 8.00 | | 9.00 | | | 7.00 | | | | 8.00 | | | | 9.50 | | | | 8.50 | | | | 8.00 | | | | 8.00 | | 8.00 | | | | | | |  | | | |
|  |  |  | | | | | |  | |  | | | |  | | | |  | | | |  | | | |  | | | |  | | | |  |  | | | | | |  | | |  | | | |
| **Ladder lung** | **NSC** | | | | | | | | | | | | | | | | | | | | | | | | | | | | | | | | | |  | | | | | |  | | |  | | | |
| 0w | 6.50 | | | 7.00 | | 7.50 | | | | 8.00 | | 9.00 | | | 6.00 | | | | 5.50 | | | | 6.00 | | | | 8.00 | | | | 6.50 | | | |  | |  | | | | |  | | |  |  |  |
| 1w | 8.00 | | | 8.67 | | 9.50 | | | | 7.00 | | 9.00 | | | 7.00 | | | | 8.00 | | | | 8.00 | | | | 7.00 | | | | 9.00 | | | |  | |  | | | | |  | | |  |  |  |
| 2w | 7.00 | | | 7.00 | | 10.00 | | | | 7.00 | | 9.50 | | | 7.00 | | | | 9.50 | | | | 9.50 | | | | 8.50 | | | | 8.00 | | | |  | |  | | | | |  | | |  |  |  |
| 3w | 8.00 | | | 9.50 | | 9.00 | | | | 7.00 | | 9.50 | | | 9.50 | | | | 8.00 | | | | 7.00 | | | | 9.00 | | | | 7.00 | | | |  | |  | | | | |  | | |  |  |  |
| 4w | 7.67 | | | 9.00 | | 9.50 | | | | 10.00 | | 8.00 | | | 10.00 | | | | 8.50 | | | | 8.50 | | | | 8.33 | | | | 10.00 | | | |  | |  | | | | |  | | |  |  |  |
| 5w | 7.00 | | | 9.50 | | 10.00 | | | | 9.00 | | 9.50 | | | 10.00 | | | | 8.50 | | | | 8.50 | | | | 10.00 | | | | 9.00 | | | |  | |  | | | | |  | | |  |  |  |
| 6w | 7.50 | | | 10.00 | | 9.50 | | | | 9.00 | | 10.00 | | | 9.50 | | | | 10.00 | | | | 9.00 | | | | 9.50 | | | | 8.00 | | | |  | |  | | | | |  | | |  |  |  |
| 7w | 8.50 | | | 10.00 | | 9.50 | | | | 9.00 | | 9.50 | | | 9.50 | | | | 9.75 | | | | 8.75 | | | | 9.75 | | | | 8.25 | | | |  | |  | | | | |  | | |  |  |  |
| 8w | 9.50 | | | 10.00 | | 9.50 | | | | 9.00 | | 9.00 | | | 9.50 | | | | 9.50 | | | | 8.50 | | | | 10.00 | | | | 8.50 | | | |  | |  | | | | |  | | |  |  |  |
|  |  |  | | | | | |  | |  | | | |  | | | |  | | | |  | | | |  | | | |  | | | |  |  | | | | | |  | | |  | | | |
| **Ladder lung** | **NSC+Exo** | | | | | | | | | | | | | | | | | | | | | | | | | | | | | | | | | | | | | | | | | | |  | | | |
| 0w | 7.50 | | | 8.50 | | 6.00 | | | | 7.50 | | 9.00 | | | 6.50 | | | | 5.50 | | | | 6.50 | | | | 9.50 | | | | 3.50 | | | | 7.00 | 6.00 | | | | | | | |  | | | |
| 1w | 8.50 | | | 8.50 | | 8.00 | | | | 8.00 | | 9.50 | | | 7.33 | | | | 8.50 | | | | 10.00 | | | | 9.00 | | | | 9.00 | | | | 7.33 | 8.50 | | | | | | | |  | | | |
| 2w | 9.50 | | | 10.00 | | 8.50 | | | | 9.50 | | 9.50 | | | 8.50 | | | | 9.00 | | | | 10.00 | | | | 9.00 | | | | 9.00 | | | | 9.50 | 8.50 | | | | | | | |  | | | |
| 3w | 9.00 | | | 9.50 | | 8.00 | | | | 9.00 | | 10.00 | | | 9.50 | | | | 8.33 | | | | 10.00 | | | | 10.00 | | | | 10.00 | | | | 10.00 | 9.00 | | | | | | | |  | | | |
| 4w | 10.00 | | | 9.50 | | 8.50 | | | | 9.50 | | 10.00 | | | 10.00 | | | | 7.67 | | | | 9.50 | | | | 10.00 | | | | 10.00 | | | | 10.00 | 9.50 | | | | | | | |  | | | |
| *Continued* | | | | | | | | | | | | | | | | | | | | | | | | | | | | | | | | | | | | | | | | | | | |  | | | |
| 5w | 9.00 | | | 9.50 | | 9.00 | | | | 8.00 | | 10.00 | | | 9.50 | | | | 9.50 | | | | 10.00 | | | | 10.00 | | | | 9.50 | | | | 9.00 |  | | | | | | | |  | | | |
| 6w | 10.00 | | | 9.50 | | 9.50 | | | | 9.00 | | 9.00 | | | 9.00 | | | | 9.50 | | | | 9.50 | | | | 10.00 | | | | 9.50 | | | | 10.00 | 9.50 | | | | | | | |  | | | |
| 7w | 9.50 | | | 9.75 | | 9.25 | | | | 9.50 | | 9.25 | | | 9.50 | | | | 9.25 | | | | 9.75 | | | | 10.00 | | | | 9.25 | | | | 10.00 | 9.75 | | | | | | | |  | | | |
| 8w | 9.00 | | | 10.00 | | 9.00 | | | | 10.00 | | 9.50 | | | 10.00 | | | | 9.00 | | | | 10.00 | | | | 10.00 | | | | 9.00 | | | | 10.00 | 10.00 | | | | | | | |  | | | |
|  |  | | | | | | | | | | | | | | | | | | | | | | | | | | | | | | | | | | | | | | | | | | |  | | | |
| **Rotarod test** | **Sham** | | | | | | | | | | | | | | | | | | | | | | | | | | | | | | | | | | | | | | | | | | |  | | | |
| 0w | 300.00 | | | 300.00 | | 300.00 | | | | 300.00 | | 300.00 | | | 300.00 | | | | 300.00 | | | | 300.00 | | | | 300.00 | | | | 260.00 | | | | 300.00 | 300.00 | | | | | | | |  | | | |
| 1w | 300.00 | | | 300.00 | | 300.00 | | | | 300.00 | | 216.67 | | | 300.00 | | | | 300.00 | | | | 300.00 | | | | 300.00 | | | | 300.00 | | | | 300.00 | 300.00 | | | | | | | |  | | | |
| 2w | 300.00 | | | 300.00 | | 300.00 | | | | 300.00 | | 300.00 | | | 300.00 | | | | 276.67 | | | | 300.00 | | | | 300.00 | | | | 300.00 | | | | 290.00 | 300.00 | | | | | | | |  | | | |
| 3w | 300.00 | | | 300.00 | | 300.00 | | | | 300.00 | | 248.33 | | | 290.00 | | | | 300.00 | | | | 300.00 | | | | 300.00 | | | | 241.33 | | | | 300.00 | 300.00 | | | | | | | |  | | | |
| 4w | 300.00 | | | 300.00 | | 300.00 | | | | 300.00 | | 274.17 | | | 295.00 | | | | 288.33 | | | | 300.00 | | | | 300.00 | | | | 270.67 | | | | 295.00 | 300.00 | | | | | | | |  | | | |
| 5w | 300.00 | | | 290.00 | | 300.00 | | | | 300.00 | | 300.00 | | | 268.33 | | | | 300.00 | | | | 300.00 | | | | 300.00 | | | | 300.00 | | | | 300.00 | 300.00 | | | | | | | |  | | | |
| 6w | 300.00 | | | 295.00 | | 300.00 | | | | 300.00 | | 287.08 | | | 281.67 | | | | 294.17 | | | | 300.00 | | | | 300.00 | | | | 285.33 | | | | 297.50 | 300.00 | | | | | | | |  | | | |
| 7w | 300.00 | | | 295.00 | | 300.00 | | | | 300.00 | | 287.08 | | | 281.67 | | | | 294.17 | | | | 300.00 | | | | 300.00 | | | | 285.33 | | | | 297.50 | 300.00 | | | | | | | |  | | | |
| 8w | 300.00 | | | 293.33 | | 300.00 | | | | 300.00 | | 291.39 | | | 277.22 | | | | 296.11 | | | | 300.00 | | | | 300.00 | | | | 290.22 | | | | 298.33 | 300.00 | | | | | | | |  | | | |
|  |  | |  | |  | | | |  | | |  | | | |  | | | |  | | | |  | | | |  | | | |  | | |  | | | |  | | | | |  | | | |
| **Rotarod test** | **Model** | | | | | | | | | | | | | | | | | | | | | | | | | | | | | | | | | | | | | | | | | | |  |  |  |  |
| 0w | 153.00 | | | 151.00 | | 224.67 | | | | 54.00 | | 251.00 | | | 48.33 | | | | 300.00 | | | | 300.00 | | | | 261.33 | | | | 42.00 | | | | 239.33 | 300.00 | | | | | | | | 224.00 | | |  |
| 1w | 153.00 | | | 165.33 | | 300.00 | | | | 300.00 | | 300.00 | | | 23.00 | | | | 28.67 | | | | 63.00 | | | | 37.33 | | | | 300.00 | | | | 47.33 | 90.00 | | | | | | | | 238.00 | | |  |
| 2w | 152.33 | | | 219.67 | | 300.00 | | | | 220.67 | | 2.67 | | | 7.00 | | | | 16.00 | | | | 140.00 | | | | 162.67 | | | | 300.00 | | | | 70.00 | 52.33 | | | | | | | | 235.33 | | |  |
| 3w | 223.67 | | | 122.67 | | 300.00 | | | | 300.00 | | 22.00 | | | 3.00 | | | | 28.33 | | | | 149.33 | | | | 165.33 | | | | 294.00 | | | | 68.33 | 71.00 | | | | | | | | 279.67 | | |  |
| 4w | 151.33 | | | 36.33 | | 300.00 | | | | 300.00 | | 52.33 | | | 110.33 | | | | 4.00 | | | | 39.33 | | | | 43.67 | | | | 300.00 | | | | 52.00 | 48.33 | | | | | | | | 243.33 | | |  |
| 5w | 297.67 | | | 74.67 | | 228.33 | | | | 300.00 | | 36.33 | | | 17.67 | | | | 26.67 | | | | 214.33 | | | | 22.33 | | | | 300.00 | | | | 63.67 | 100.00 | | | | | | | | 288.33 | | |  |
| 6w | 220.00 | | | 119.00 | | 300.00 | | | | 256.67 | | 25.67 | | | 195.67 | | | | 52.33 | | | | 124.33 | | | | 19.33 | | | | 300.00 | | | | 37.67 | 155.00 | | | | | | | | 56.00 | | |  |
| 7w | 260.00 | | | 191.33 | | 211.33 | | | | 289.17 | | 154.33 | | | 21.50 | | | | 30.08 | | | | 111.92 | | | | 103.33 | | | | 163.17 | | | | 172.58 | 63.08 | | | | | | | | 84.75 | | |  |
| 8w | 300.00 | | | 126.33 | | 300.00 | | | | 300.00 | | 35.00 | | | 11.00 | | | | 42.67 | | | | 228.33 | | | | 41.33 | | | | 292.00 | | | | 71.00 | 129.33 | | | | | | | | 129.00 | | |  |
|  |  | |  | |  | | | |  | | |  | | | |  | | | |  | | | |  | | | |  | | | |  | | |  | |  | | | | | | |  | | | |
| **Rotarod test** | **Exo** | | | | | | | | | | | | | | | | | | | | | | | | | | | | | | | | | | | | | | | | | | |  | | | |
| 0w | 263.00 | | | 245.00 | | 24.67 | | | | 25.67 | | 100.33 | | | 48.33 | | | | 72.67 | | | | 300.00 | | | | 300.00 | | | | 241.67 | | | | 300.00 | 286.33 | | | | | | | |  | | | |
| 1w | 300.00 | | | 145.67 | | 222.00 | | | | 95.00 | | 135.00 | | | 8.67 | | | | 21.67 | | | | 300.00 | | | | 286.00 | | | | 12.33 | | | | 134.33 | 129.00 | | | | | | | |  | | | |
| 2w | 300.00 | | | 120.67 | | 300.00 | | | | 259.33 | | 32.67 | | | 169.67 | | | | 20.33 | | | | 300.00 | | | | 300.00 | | | | 7.00 | | | | 283.00 | 47.00 | | | | | | | |  | | | |
| 3w | 237.33 | | | 23.67 | | 126.00 | | | | 223.33 | | 146.33 | | | 300.00 | | | | 35.00 | | | | 27.00 | | | | 278.00 | | | | 30.67 | | | | 230.00 | 89.33 | | | | | | | |  | | | |
| 4w | 279.11 | | | 11.67 | | 216.00 | | | | 192.56 | | 104.67 | | | 6.33 | | | | 20.67 | | | | 300.00 | | | | 288.00 | | | | 8.33 | | | | 256.50 | 109.17 | | | | | | | |  | | | |
| 5w | 272.15 | | | 120.67 | | 214.00 | | | | 225.07 | | 94.56 | | | 5.56 | | | | 20.33 | | | | 300.00 | | | | 288.67 | | | | 7.00 | | | | 256.50 | 99.25 | | | | | | | |  | | | |
| 6w | 262.86 | | | 120.67 | | 185.33 | | | | 213.65 | | 115.19 | | | 6.07 | | | | 20.33 | | | | 300.00 | | | | 284.89 | | | | 15.00 | | | | 247.67 | 99.25 | | | | | | | |  | | | |
| 7w | 275.63 | | | 120.67 | | 215.00 | | | | 208.81 | | 99.61 | | | 5.94 | | | | 20.50 | | | | 300.00 | | | | 288.33 | | | | 242.31 | | | | 256.50 | 104.21 | | | | | | | |  | | | |
| *Continued* | | | | | | | | | | | | | | | | | | | | | | | | | | | | | | | | | | | | | | | | | | | |  | | | |
| 8w | 270.21 | | | 120.67 | | 204.78 | | | | 215.85 | | 103.12 | | | 5.86 | | | | 241.24 | | | | 56.67 | | | | 287.30 | | | | 300.00 | | | | 253.56 | 100.90 | | | | | | | |  | | | |

| **Rotarod test** | | **NSC** | | | | | | | | | | | | | | | | | | | | | | | | | | | | | | | | |  | | |  | | | |  | | | |  |  |
| --- | --- | --- | --- | --- | --- | --- | --- | --- | --- | --- | --- | --- | --- | --- | --- | --- | --- | --- | --- | --- | --- | --- | --- | --- | --- | --- | --- | --- | --- | --- | --- | --- | --- | --- | --- | --- | --- | --- | --- | --- | --- | --- | --- | --- | --- | --- | --- |
| 0w | 300.00 | | 141.00 | | | | 120.00 | | | | 113.00 | | | | 124.33 | | 300.00 | | | 130.00 | | | | 241.00 | | | | 225.00 | | | 300.00 | | | |  |  | | | | |  | | |  |  |  |  |
| 1w | 300.00 | | 244.67 | | | | 239.33 | | | | 219.67 | | | | 96.33 | | 300.00 | | | 38.33 | | | | 300.00 | | | | 290.00 | | | 225.00 | | | |  |  | | | | |  | | |  |  |  |  |
| 2w | 183.33 | | 170.00 | | | | 300.00 | | | | 178.33 | | | | 244.00 | | 300.00 | | | 249.00 | | | | 300.00 | | | | 152.67 | | | 171.67 | | | |  |  | | | | |  | | |  |  |  |  |
| 3w | 300.00 | | 300.00 | | | | 300.00 | | | | 250.00 | | | | 300.00 | | 92.67 | | | 111.67 | | | | 276.67 | | | | 256.00 | | | 73.33 | | | |  |  | | | | |  | | |  |  |  |  |
| 4w | 212.67 | | 249.33 | | | | 292.67 | | | | 172.67 | | | | 300.00 | | 300.00 | | | 122.67 | | | | 300.00 | | | | 45.00 | | | 65.33 | | | |  |  | | | | |  | | |  |  |  |  |
| 5w | 38.33 | | 300.00 | | | | 277.67 | | | | 235.83 | | | | 279.67 | | 289.00 | | | 30.67 | | | | 300.00 | | | | 61.00 | | | 239.00 | | | |  |  | | | | |  | | |  |  |  |  |
| 6w | 287.67 | | 300.00 | | | | 300.00 | | | | 136.33 | | | | 300.00 | | 275.00 | | | 300.00 | | | | 67.67 | | | | 300.00 | | | 62.67 | | | |  |  | | | | |  | | |  |  |  |  |
| 7w | 16.17 | | 300.00 | | | | 258.33 | | | | 284.33 | | | | 300.00 | | 287.50 | | | 173.00 | | | | 73.33 | | | | 300.00 | | | 158.83 | | | |  |  | | | | |  | | |  |  |  |  |
| 8w | 12.00 | | 300.00 | | | | 216.67 | | | | 171.67 | | | | 300.00 | | 300.00 | | | 173.67 | | | | 79.00 | | | | 300.00 | | | 290.00 | | | |  |  | | | | |  | | |  |  |  |  |
|  | |  | |  | | | |  | | | |  | | | |  | | |  | | | |  | | | |  | | |  | | | |  |  | | |  | | | |  | | | |  |  |
| **Rotarod test** | | **NSC+Exo** | | | | | | | | | | | | | | | | | | | | | | | | | | | | | | | | | | | | | | | |  | | | |  |  |
| 0w | | 261.33 | | | 217.33 | | | | 224.00 | | | | 179.67 | | | 188.33 | | 300.00 | | | | 23.67 | | | | 300.00 | | | 300.00 | | | 122.67 | | | 122.00 | | 53.00 | | | | |  | | | |  |  |
| 1w | | 300.00 | | | 168.33 | | | | 235.33 | | | | 161.67 | | | 239.33 | | 224.33 | | | | 55.67 | | | | 300.00 | | | 300.00 | | | 107.67 | | | 300.00 | | 266.00 | | | | |  | | | |  |  |
| 2w | | 300.00 | | | 78.33 | | | | 263.67 | | | | 300.00 | | | 300.00 | | 300.00 | | | | 181.33 | | | | 300.00 | | | 300.00 | | | 60.00 | | | 245.33 | | 160.67 | | | | |  | | | |  |  |
| 3w | | 237.33 | | | 170.00 | | | | 275.33 | | | | 225.67 | | | 300.00 | | 300.00 | | | | 84.33 | | | | 300.00 | | | 300.00 | | | 300.00 | | | 256.00 | | 300.00 | | | | |  | | | |  |  |
| 4w | | 300.00 | | | 46.67 | | | | 300.00 | | | | 153.33 | | | 259.33 | | 300.00 | | | | 69.67 | | | | 239.00 | | | 247.67 | | | 198.00 | | | 268.67 | | 300.00 | | | | |  | | | |  |  |
| 5w | | 300.00 | | | 156.67 | | | | 300.00 | | | | 300.00 | | | 300.00 | | 300.00 | | | | 126.33 | | | | 300.00 | | | 300.00 | | | 73.00 | | | 300.00 | | 300.00 | | | | |  | | | |  |  |
| 6w | | 300.00 | | | 96.33 | | | | 300.00 | | | | 300.00 | | | 300.00 | | 300.00 | | | | 117.67 | | | | 300.00 | | | 300.00 | | | 102.00 | | | 300.00 | | 300.00 | | | | |  | | | |  |  |
| 7w | | 300.00 | | | 57.17 | | | | 300.00 | | | | 300.00 | | | 269.83 | | 287.17 | | | | 108.17 | | | | 300.00 | | | 268.33 | | | 300.00 | | | 266.50 | | 300.00 | | | | |  | | | |  |  |
| 8w | | 300.00 | | | 65.00 | | | | 300.00 | | | | 300.00 | | | 239.67 | | 274.33 | | | | 98.67 | | | | 300.00 | | | 236.67 | | | 260.00 | | | 233.00 | | 300.00 | | | | |  | | | |  |  |
|  | |  | | |  | | | |  | | | |  | | |  | |  | | | |  | | | |  | | |  | | |  | | |  | |  | | | | |  | | | |  |  |
| **mNss** | | **Sham** | | | | | | | | | | | | | | | | | | | | | | | | | | | | | | | | | | | | | | | |  | | | |  |  |
| 0w | | 0 | | | 0 | | | | 0 | | | | 0 | | | 1 | | 0 | | | | 0 | | | | 0 | | | 0 | | | 0 | | | 0 | | 0 | | | | |  | | | |  |  |
| 1w | | 0 | | | 0 | | | | 0 | | | | 0 | | | 2 | | 1 | | | | 0 | | | | 0 | | | 0 | | | 0 | | | 0 | | 0 | | | | |  | | | |  |  |
| 2w | | 0 | | | 0 | | | | 0 | | | | 0 | | | 1 | | 0 | | | | 0 | | | | 0 | | | 0 | | | 0 | | | 0 | | 0 | | | | |  | | | |  |  |
| 3w | | 0 | | | 0 | | | | 0 | | | | 0 | | | 0 | | 0 | | | | 0 | | | | 1 | | | 0 | | | 0 | | | 0 | | 0 | | | | |  | | | |  |  |
| 4w | | 0 | | | 0 | | | | 0 | | | | 0 | | | 0 | | 0 | | | | 0 | | | | 1 | | | 0 | | | 0 | | | 0 | | 0 | | | | |  | | | |  |  |
| 5w | | 0 | | | 0 | | | | 0 | | | | 0 | | | 0 | | 0 | | | | 0 | | | | 0 | | | 0 | | | 0 | | | 0 | | 0 | | | | |  | | | |  |  |
| 6w | | 0 | | | 0 | | | | 0 | | | | 0 | | | 0 | | 0 | | | | 0 | | | | 0 | | | 0 | | | 0 | | | 0 | | 0 | | | | |  | | | |  |  |
| 7w | | 0 | | | 0 | | | | 0 | | | | 0 | | | 0 | | 0 | | | | 0 | | | | 0 | | | 1 | | | 0 | | | 0 | | 0 | | | | |  | | | |  |  |
| 8w | | 0 | | | 0 | | | | 0 | | | | 0 | | | 1 | | 2 | | | | 0 | | | | 0 | | | 0 | | | 0 | | | 0 | | 0 | | | | |  | | | |  |  |
|  | |  | |  | |  | | | |  | | | |  | | |  | | | |  | | | |  | | | |  | | | |  | |  | | | |  | | |  | | | | |  |
|  | |  | |  | |  | | | |  | | | |  | | |  | | | |  | | | |  | | | |  | | | |  | |  | | | |  | | |  | | | | |  |
| **mNss** | | **Model** | | | | | | | | | | | | | | | | | | | | | | | | | | | | | | | | | | | | | | | |  |  |  |  |  |  |
| 0w | | 6 | | | 9 | | | | 4 | | | | 9 | | | 9 | | 6 | | | | 5 | | | | 8 | | | 8 | | | 9 | | | 6 | | 9 | | | | | 3 | | |  |  |  |
| 1w | | 6 | | | 10 | | | | 5 | | | | 9 | | | 9 | | 3 | | | | 4 | | | | 7 | | | 8 | | | 8 | | | 4 | | 9 | | | | | 4 | | |  |  |  |
| 2w | | 5 | | | 9 | | | | 6 | | | | 8 | | | 9 | | 4 | | | | 3 | | | | 6 | | | 8 | | | 8 | | | 4 | | 8 | | | | | 5 | | |  |  |  |
| 3w | | 6 | | | 8 | | | | 6 | | | | 7 | | | 8 | | 6 | | | | 3 | | | | 5 | | | 7 | | | 8 | | | 4 | | 9 | | | | | 5 | | |  |  |  |
| 4w | | 4 | | | 8 | | | | 5 | | | | 9 | | | 8 | | 7 | | | | 3 | | | | 5 | | | 6 | | | 7 | | | 4 | | 8 | | | | | 4 | | |  |  |  |
| 5w | | 5 | | | 8 | | | | 6 | | | | 8 | | | 8 | | 5 | | | | 3 | | | | 5 | | | 5 | | | 6 | | | 4 | | 6 | | | | | 3 | | |  |  |  |
| 6w | | 3 | | | 8 | | | | 7 | | | | 7 | | | 8 | | 6 | | | | 3 | | | | 6 | | | 6 | | | 5 | | | 3 | | 6 | | | | | 7 | | |  |  |  |
| 7w | | 5 | | | 6 | | | | 6 | | | | 7 | | | 8 | | 5 | | | | 2 | | | | 7 | | | 6 | | | 6 | | | 3 | | 6 | | | | | 4 | | |  |  |  |
| 8w | | 3 | | | 6 | | | | 4 | | | | 8 | | | 7 | | 5 | | | | 3 | | | | 5 | | | 5 | | | 4 | | | 3 | | 4 | | | | | 4 | | |  |  |  |
|  | |  | |  | |  | | | |  | | | |  | | |  | | | |  | | | |  | | | |  | | | |  | |  | | |  | | | |  | | | | |  |
| **mNss** | | **Exo** | | | | | | | | | | | | | | | | | | | | | | | | | | | | | | | | | | | | | | | |  | | | |  |  |
| 0w | | 6 | | | 7 | | | | 9 | | | | 7 | | | 8 | | 9 | | | | 9 | | | | 5 | | | 5 | | | 3 | | | 3 | | 10 | | | | |  | | | | |  |
| 1w | | 6 | | | 7 | | | | 8 | | | | 8 | | | 7 | | 9 | | | | 9 | | | | 4 | | | 4 | | | 4 | | | 7 | | 4 | | | | |  | | | | |  |
| 2w | | 6 | | | 6 | | | | 7 | | | | 6 | | | 7 | | 8 | | | | 9 | | | | 3 | | | 4 | | | 6 | | | 6 | | 7 | | | | |  | | | | |  |
| 3w | | 4 | | | 9 | | | | 6 | | | | 7 | | | 6 | | 9 | | | | 8 | | | | 3 | | | 3 | | | 7 | | | 5 | | 5 | | | | |  | | | | |  |
| 4w | | 5 | | | 4 | | | | 6 | | | | 5 | | | 4 | | 8 | | | | 9 | | | | 3 | | | 4 | | | 8 | | | 6 | | 6 | | | | |  | | | | |  |
| 5w | | 5 | | | 4 | | | | 5 | | | | 4 | | | 5 | | 8 | | | | 9 | | | | 3 | | | 3 | | | 9 | | | 8 | | 6 | | | | |  | | | | |  |
| 6w | | 4 | | | 4 | | | | 3 | | | | 3 | | | 3 | | 7 | | | | 8 | | | | 3 | | | 3 | | | 9 | | | 6 | | 5 | | | | |  | | | | |  |
| 7w | | 5 | | | 4 | | | | 4 | | | | 3 | | | 4 | | 6 | | | | 7 | | | | 2 | | | 3 | | | 5 | | | 6 | | 5 | | | | |  | | | | |  |
| 8w | | 3 | | | 4 | | | | 3 | | | | 3 | | | 4 | | 6 | | | | 8 | | | | 3 | | | 3 | | | 8 | | | 5 | | 4 | | | | |  | | | | |  |
|  | |  | |  | | | |  | |  | | | |  | | |  | | | |  | | | |  | | | |  | | | |  | |  | | |  | | | |  | | | | |  |
| **mNss** | | **NSC** | | | | | | | | | | | | | | | | | | | | | | | | | | | | | | | | |  | | |  | | | |  | | | |  |  |
| 0w | | 7 | | | 6 | | | | 3 | | | | 9 | | | 7 | | 7 | | | | 9 | | | | 6 | | | 7 | | | 6 | | |  | | | | |  | | |  | | | | |
| 1w | | 5 | | | 5 | | | | 3 | | | | 3 | | | 4 | | 5 | | | | 8 | | | | 5 | | | 4 | | | 3 | | |  | | | | |  | | |  | | | | |
| 2w | | 4 | | | 4 | | | | 1 | | | | 3 | | | 3 | | 3 | | | | 6 | | | | 5 | | | 4 | | | 2 | | |  | | | | |  | | |  | | | | |
| 3w | | 5 | | | 3 | | | | 1 | | | | 2 | | | 2 | | 4 | | | | 5 | | | | 4 | | | 3 | | | 3 | | |  | | | | |  | | |  | | | | |
| 4w | | 3 | | | 3 | | | | 2 | | | | 3 | | | 2 | | 4 | | | | 5 | | | | 3 | | | 2 | | | 2 | | |  | | | | |  | | |  | | | | |
| 5w | | 6 | | | 2 | | | | 1 | | | | 2 | | | 2 | | 2 | | | | 4 | | | | 3 | | | 3 | | | 3 | | |  | | | | |  | | |  | | | | |
| 6w | | 5 | | | 2 | | | | 2 | | | | 1 | | | 1 | | 2 | | | | 3 | | | | 2 | | | 1 | | | 4 | | |  | | | | |  | | |  | | | | |
| 7w | | 5 | | | 0 | | | | 2 | | | | 1 | | | 1 | | 2 | | | | 2 | | | | 4 | | | 2 | | | 2 | | |  | | | | |  | | |  | | | | |
| 8w | | 6 | | | 0 | | | | 2 | | | | 1 | | | 2 | | 1 | | | | 3 | | | | 3 | | | 0 | | | 2 | | |  | | | | |  | | |  | | | | |
|  | |  | |  | | | |  | |  | | | |  | | |  | | | |  | | | |  | | | |  | | | |  | |  | | |  | | | |  | | | | |  |
| **mNss** | | **NSC+Exo** | | | | | | | | | | | | | | | | | | | | | | | | | | | | | | | | | | | | | | | |  | | | |  |  |
| 0w | | 7 | | | 6 | | | | 7 | | | | 9 | | | 7 | | 7 | | | | 9 | | | | 6 | | | 7 | | | 8 | | | 7 | | 7 | | | | |  | | | | |  |
| 1w | | 3 | | | 4 | | | | 3 | | | | 6 | | | 4 | | 5 | | | | 7 | | | | 3 | | | 5 | | | 7 | | | 3 | | 3 | | | | |  | | | | |  |
| 2w | | 2 | | | 3 | | | | 3 | | | | 4 | | | 4 | | 4 | | | | 5 | | | | 2 | | | 3 | | | 7 | | | 3 | | 4 | | | | |  | | | | |  |
| 3w | | 3 | | | 3 | | | | 2 | | | | 3 | | | 1 | | 3 | | | | 4 | | | | 1 | | | 2 | | | 5 | | | 1 | | 1 | | | | |  | | | | |  |
| 4w | | 2 | | | 3 | | | | 1 | | | | 3 | | | 1 | | 1 | | | | 4 | | | | 1 | | | 1 | | | 5 | | | 1 | | 1 | | | | |  | | | | |  |
| *Continued* | | | | | | | | | | | | | | | | | | | | | | | | | | | | | | | | | | | | | | | | | |  | | | | |  |
| 5w | | 2 | | | 2 | | | | 2 | | | | 3 | | | 1 | | 1 | | | | 3 | | | | 0 | | | 2 | | | 2 | | | 1 | | 2 | | | | |  | | | | |  |
| 6w | | 0 | | | 2 | | | | 1 | | | | 1 | | | 1 | | 2 | | | | 2 | | | | 1 | | | 1 | | | 3 | | | 0 | | 1 | | | | |  | | | | |  |
| 7w | | 1 | | | 3 | | | | 1 | | | | 0 | | | 0 | | 2 | | | | 2 | | | | 1 | | | 1 | | | 3 | | | 1 | | 1 | | | | |  | | | | |  |
| 8w | | 1 | | | 2 | | | | 0 | | | | 0 | | | 1 | | 2 | | | | 2 | | | | 0 | | | 0 | | | 2 | | | 1 | | 0 | | | | |  | | | | |  |
