# Supplementary material for: NSC-derived exosomes enhance therapeutic effects of NSC transplantation on cerebral ischemia in mice
Source: eLife. 2023 Apr 27;12:e84493. doi: 10.7554/eLife.84493 (PMC10139690; doi:10.7554/eLife.84493)
Supplement: Figure 2—source data 1. [file elife-84493-fig2-data1.zip › Figure 2-source data 1/Figure 2-source data 1.docx]

| **Figure 2B-Resource data: Defect volume data** | | | | |
| --- | --- | --- | --- | --- |
| Sham | Model | Exo | NSC | NSC+Exo |
| 0.1 | 21.02 | 22.89 | 18.89 | 9.63 |
| 0 | 22.59 | 23.15 | 8.99 | 8.31 |
| 0.5 | 22.67 | 17.27 | 14.84 | 9.17 |
| 0.5 | 23.33 | 18.00 | 14.56 | 3.16 |

| **Figure 2D-Resource data: dendritic length:** | | | | | | | | | | | | |
| --- | --- | --- | --- | --- | --- | --- | --- | --- | --- | --- | --- | --- |
| sham | 1.00 | | 2.00 | | 3.00 | | 4.00 | | 5.00 | | 6.00 | |
|  | 30.00 | 13.05 | 30.00 | 10.59 | 30.00 | 10.06 | 30.00 | 15.99 | 30.00 | 8.00 | 30.00 | 3.00 |
|  | 40.00 | 19.92 | 40.00 | 19.76 | 40.00 | 19.51 | 40.00 | 19.04 | 40.00 | 18.00 | 40.00 | 12.99 |
|  | 50.00 | 21.69 | 50.00 | 23.81 | 50.00 | 22.66 | 50.00 | 22.87 | 50.00 | 21.00 | 50.00 | 15.05 |
|  | 60.00 | 22.81 | 60.00 | 24.66 | 60.00 | 27.15 | 60.00 | 25.21 | 60.00 | 19.00 | 60.00 | 22.90 |
|  | 70.00 | 24.17 | 70.00 | 23.83 | 70.00 | 30.14 | 70.00 | 30.79 | 70.00 | 19.00 | 70.00 | 22.12 |
|  | 80.00 | 24.99 | 80.00 | 22.41 | 80.00 | 28.98 | 80.00 | 35.13 | 80.00 | 17.00 | 80.00 | 17.91 |
|  | 90.00 | 24.30 | 90.00 | 20.95 | 90.00 | 25.14 | 90.00 | 30.96 | 90.00 | 18.00 | 90.00 | 21.04 |
|  | 100.00 | 21.76 | 100.00 | 19.58 | 100.00 | 21.98 | 100.00 | 22.00 |  |  | 100.00 | 19.99 |
|  | 110.00 | 17.90 | 110.00 | 18.12 | 110.00 | 19.83 |  |  |  |  | 110.00 | 14.00 |
|  | 120.00 | 13.86 | 120.00 | 16.29 | 120.00 | 14.63 |  |  |  |  |  |  |
|  | 130.00 | 10.52 | 130.00 | 13.90 | 130.00 | 8.92 |  |  |  |  |  |  |
|  | 140.00 | 7.04 | 140.00 | 11.00 |  |  |  |  |  |  |  |  |
|  | *Continued* | | | | | | | | | | | |
|  |  |  | 150.00 | 7.95 |  |  |  |  |  |  |  |  |
|  |  |  | 160.00 | 5.40 |  |  |  |  |  |  |  |  |
|  |  |  | 170.00 | 3.96 |  |  |  |  |  |  |  |  |
|  |  |  | 180.00 | 3.68 |  |  |  |  |  |  |  |  |
|  |  |  | 190.00 | 3.11 |  |  |  |  |  |  |  |  |
|  |  | 634.00 |  | 1206.00 |  | 1171.00 |  | 800.00 |  | 1178.00 |  | 1014.00 |
|  |  |  |  |  |  |  |  |  |  |  |  |  |
| model | 1.00 | | 2.00 | | 3.00 | | 4.00 | | 5.00 | | 6.00 | |
|  | 30.00 | 7.03 | 30.00 | 3.82 | 30.00 | 4.69 | 30.00 | 6.98 | 30.00 | 5.00 | 30.00 | 8.00 |
|  | 40.00 | 6.86 | 40.00 | 10.69 | 40.00 | 4.82 | 40.00 | 4.17 | 40.00 | 3.00 | 40.00 | 6.00 |
|  | 50.00 | 8.25 | 50.00 | 11.06 | 50.00 | 6.90 | 50.00 | 7.50 | 50.00 | 4.00 | 50.00 | 5.00 |
|  | 60.00 | 7.88 | 60.00 | 9.56 | 60.00 | 10.93 | 60.00 | 4.84 | 60.00 | 3.00 | 60.00 | 1.00 |
|  | 70.00 | 7.78 | 70.00 | 8.56 | 70.00 | 15.43 | 70.00 | 2.16 |  |  | 70.00 | 2.00 |
|  | 80.00 | 9.41 | 80.00 | 8.46 | 80.00 | 18.77 | 80.00 | 2.50 |  |  |  |  |
|  | 90.00 | 9.70 | 90.00 | 8.72 | 90.00 | 20.04 | 90.00 | 1.83 |  |  |  |  |
|  | 100.00 | 4.11 | 100.00 | 8.85 | 100.00 | 19.35 | 100.00 | 2.02 |  |  |  |  |
|  | 110.00 | 2.99 | 110.00 | 8.99 | 110.00 | 17.61 |  |  |  |  |  |  |
|  |  |  | 120.00 | 9.16 | 120.00 | 15.94 |  |  |  |  |  |  |
|  |  |  | 130.00 | 6.14 | 130.00 | 14.97 |  |  |  |  |  |  |
|  |  |  |  |  | 140.00 | 14.37 |  |  |  |  |  |  |
|  |  |  |  |  | 150.00 | 13.14 |  |  |  |  |  |  |
|  |  |  |  |  | 160.00 | 11.17 |  |  |  |  |  |  |
|  | *Continued* | | | | | | | | | | | |
|  |  |  |  |  | 170.00 | 12.89 |  |  |  |  |  |  |
|  |  | 477.00 |  | 395.00 |  | 1020.00 |  | 120.00 |  | 110.00 |  | 115.00 |
|  |  |  |  |  |  |  |  |  |  |  |  |  |
| Exo | 1.00 | | 2.00 | | 3.00 | | 4.00 | | 5.00 | | 6.00 | |
|  | 30.00 | 5.00 | 30.00 | 4.00 | 30.00 | 4.00 | 30.00 | 3.00 | 30.00 | 5.00 | 30.00 | 4.00 |
|  | 40.00 | 11.00 | 40.00 | 3.00 | 40.00 | 5.00 | 40.00 | 5.00 | 40.00 | 2.00 | 40.00 | 4.00 |
|  | 50.00 | 10.00 | 50.00 | 1.00 | 50.00 | 6.00 | 50.00 | 2.00 | 50.00 | 2.00 | 50.00 | 5.00 |
|  | 60.00 | 8.00 |  |  | 60.00 | 7.00 | 60.00 | 3.00 | 60.00 | 2.00 | 60.00 | 3.00 |
|  | 70.00 | 6.00 |  |  | 70.00 | 6.00 |  |  | 70.00 | 2.00 | 70.00 | 1.00 |
|  | 80.00 | 5.00 |  |  |  |  |  |  | 80.00 | 4.00 | 80.00 | 1.00 |
|  | 90.00 | 4.00 |  |  |  |  |  |  | 90.00 | 2.00 |  |  |
|  | 100.00 | 5.00 |  |  |  |  |  |  |  |  |  |  |
|  | 110.00 | 3.00 |  |  |  |  |  |  |  |  |  |  |
|  |  | 541.00 |  | 60.00 |  | 145.00 |  | 125.00 |  | 185.00 |  | 185.00 |
|  |  |  |  |  |  |  |  |  |  |  |  |  |
| NSC | 1.00 | | 2.00 | | 3.00 | | 4.00 | | 5.00 | | 6.00 | |
|  | 30.00 | 12.20 | 30.00 | 12.04 | 30.00 | 12.16 | 30.00 | 7.29 | 30.00 | 6.70 | 30.00 | 7.68 |
|  | 40.00 | 6.86 | 40.00 | 9.63 | 40.00 | 14.10 | 40.00 | 10.73 | 40.00 | 7.96 | 40.00 | 10.39 |
|  | 50.00 | 6.51 | 50.00 | 8.26 | 50.00 | 15.05 | 50.00 | 11.52 | 50.00 | 7.46 | 50.00 | 9.73 |
|  | 60.00 | 7.49 | 60.00 | 9.22 | 60.00 | 18.65 | 60.00 | 10.97 | 60.00 | 6.43 | 60.00 | 8.83 |
|  | 70.00 | 8.59 | 70.00 | 11.22 | 70.00 | 22.22 | 70.00 | 10.02 | 70.00 | 5.49 | 70.00 | 8.85 |
|  | 80.00 | 9.37 | 80.00 | 12.59 | 80.00 | 23.67 | 80.00 | 9.26 | 80.00 | 4.88 | 80.00 | 9.81 |
|  | *Continued* | | | | | | | | | | | |
|  | 90.00 | 9.46 | 90.00 | 12.48 | 90.00 | 23.67 | 90.00 | 8.93 | 90.00 | 4.61 | 90.00 | 11.19 |
|  | 100.00 | 8.60 | 100.00 | 11.23 | 100.00 | 24.54 | 100.00 | 9.02 | 100.00 | 4.60 | 100.00 | 12.33 |
|  | 110.00 | 7.16 | 110.00 | 9.95 | 110.00 | 27.50 | 110.00 | 9.34 | 110.00 | 4.72 | 110.00 | 12.75 |
|  | 120.00 | 6.52 | 120.00 | 9.56 | 120.00 | 25.00 | 120.00 | 9.64 | 120.00 | 4.86 | 120.00 | 12.22 |
|  | 130.00 | 8.11 | 130.00 | 9.96 | 130.00 | 15.00 | 130.00 | 9.72 | 130.00 | 4.93 | 130.00 | 10.84 |
|  | 140.00 | 10.13 | 140.00 | 9.72 | 140.00 | 17.17 | 140.00 | 9.47 | 140.00 | 4.92 | 140.00 | 8.95 |
|  |  |  | 150.00 | 7.19 | 150.00 | 13.29 | 150.00 | 8.94 | 150.00 | 4.83 | 150.00 | 7.04 |
|  |  |  | 160.00 | 3.96 |  |  | 160.00 | 8.27 | 160.00 | 4.68 | 160.00 | 5.59 |
|  |  |  |  |  |  |  | 170.00 | 7.54 | 170.00 | 4.52 | 170.00 | 4.96 |
|  |  |  |  |  |  |  | 180.00 | 6.45 | 180.00 | 4.40 | 180.00 | 5.21 |
|  |  |  |  |  |  |  | 190.00 | 3.90 | 190.00 | 4.32 | 190.00 | 6.12 |
|  |  |  |  |  |  |  |  |  | 200.00 | 4.30 | 200.00 | 7.11 |
|  |  |  |  |  |  |  |  |  | 210.00 | 4.31 | 210.00 | 7.37 |
|  |  |  |  |  |  |  |  |  | 220.00 | 4.32 | 220.00 | 6.12 |
|  |  |  |  |  |  |  |  |  | 230.00 | 4.27 | 230.00 | 2.94 |
|  |  |  |  |  |  |  |  |  | 240.00 | 4.12 | 240.00 | 0.00 |
|  |  | 1336.00 |  | 1120.00 |  | 535.00 |  | 715.00 |  | 980.00 |  | 1025.00 |
|  |  |  |  |  |  |  |  |  |  |  |  |  |
| NE | 1.00 | | 2.00 | | 3.00 | | 4.00 | | 5.00 | | 6.00 | |
|  | 20.00 | 5.70 | 20.00 | 7.31 | 30.00 | 10.22 | 30.00 | 3.00 | 20.00 | 8.02 | 30.00 | 8.72 |
|  | 30.00 | 8.30 | 30.00 | 8.33 | 40.00 | 16.84 | 40.00 | 7.00 | 30.00 | 12.92 | 40.00 | 9.10 |
|  | 40.00 | 7.09 | 40.00 | 13.27 | 50.00 | 19.45 | 50.00 | 11.00 | 40.00 | 16.89 | 50.00 | 5.07 |
|  | *Continued* | | | | | | | | | | | |
|  | 50.00 | 7.49 | 50.00 | 18.89 | 60.00 | 21.29 | 60.00 | 16.00 | 50.00 | 17.13 | 60.00 | 5.58 |
|  | 60.00 | 10.22 | 60.00 | 23.37 | 70.00 | 23.66 | 70.00 | 20.00 | 60.00 | 18.03 | 70.00 | 10.07 |
|  | 70.00 | 13.15 | 70.00 | 25.89 | 80.00 | 26.71 | 80.00 | 24.00 | 70.00 | 22.32 | 80.00 | 14.47 |
|  | 80.00 | 13.86 | 80.00 | 26.32 | 90.00 | 29.98 | 90.00 | 25.00 | 80.00 | 18.00 | 90.00 | 15.45 |
|  | 90.00 | 11.52 | 90.00 | 24.96 | 100.00 | 32.81 | 100.00 | 24.00 | 90.00 | 11.00 | 100.00 | 12.48 |
|  | 100.00 | 7.59 | 100.00 | 22.35 | 110.00 | 34.61 | 110.00 | 22.00 | 100.00 | 11.00 | 110.00 | 7.80 |
|  | 110.00 | 4.90 | 110.00 | 19.09 | 120.00 | 34.97 | 120.00 | 20.00 | 110.00 | 11.00 | 120.00 | 4.68 |
|  | 120.00 | 5.25 | 120.00 | 15.74 | 130.00 | 33.75 | 130.00 | 19.00 | 120.00 | 7.00 | 130.00 | 4.96 |
|  | 130.00 | 5.95 | 130.00 | 12.76 | 140.00 | 31.07 | 140.00 | 19.00 |  |  | 140.00 | 7.12 |
|  |  |  | 140.00 | 10.44 | 150.00 | 27.25 | 150.00 | 17.00 |  |  | 150.00 | 6.65 |
|  |  |  | 150.00 | 8.91 | 160.00 | 22.77 | 160.00 | 12.00 |  |  | 160.00 | 0.86 |
|  |  |  | 160.00 | 8.16 | 170.00 | 18.14 | 170.00 | 16.00 |  |  |  |  |
|  |  |  | 170.00 | 8.05 | 180.00 | 13.87 | 180.00 | 7.00 |  |  |  |  |
|  |  |  | 180.00 | 8.36 | 190.00 | 10.36 |  |  |  |  |  |  |
|  |  |  | 190.00 | 8.83 | 200.00 | 7.88 |  |  |  |  |  |  |
|  |  |  | 200.00 | 9.21 | 210.00 | 6.51 |  |  |  |  |  |  |
|  |  |  | 210.00 | 9.31 | 220.00 | 6.14 |  |  |  |  |  |  |
|  |  |  | 220.00 | 9.05 | 230.00 | 6.52 |  |  |  |  |  |  |
|  |  |  | 230.00 | 8.44 | 240.00 | 7.30 |  |  |  |  |  |  |
|  |  |  | 240.00 | 7.62 | 250.00 | 8.06 |  |  |  |  |  |  |
|  |  |  | 250.00 | 6.81 | 260.00 | 8.44 |  |  |  |  |  |  |
|  |  |  | 260.00 | 6.26 | 270.00 | 8.17 |  |  |  |  |  |  |
|  | *Continued* | | | | | | | | | | | |
|  |  |  | 270.00 | 6.19 | 280.00 | 7.18 |  |  |  |  |  |  |
|  |  |  | 280.00 | 6.59 | 290.00 | 5.59 |  |  |  |  |  |  |
|  |  |  | 290.00 | 7.09 | 300.00 | 3.77 |  |  |  |  |  |  |
|  |  |  | 300.00 | 6.74 | 310.00 | 2.21 |  |  |  |  |  |  |
|  |  |  | 310.00 | 3.65 | 320.00 | 1.42 |  |  |  |  |  |  |
|  |  |  | 310.00 | 4.00 | 330.00 | 1.63 |  |  |  |  |  |  |
|  |  | 690.00 |  | 1300.00 |  | 1020.00 |  | 850.00 |  | 645.00 |  | 1200.00 |

| **Figure 2E-Resource data: dendritic spines:** | | | | | | |
| --- | --- | --- | --- | --- | --- | --- |
| sham | 27 | 20 | 20 | 28 | 27 | 25 |
| model | 0 | 0 | 3 | 4 | 4 | 2 |
| exo | 7 | 3 | 2 | 2 | 3 | 3 |
| nsc | 12 | 11 | 3 | 14 | 15 | 7 |
| Nsc+Exo | 26 | 21 | 29 | 5 | 20 | 18 |

| **Figure 2F-Resource data: Intersections number:** | | | | | | |
| --- | --- | --- | --- | --- | --- | --- |
|  | **Sham** | | | | | |
| 30.00 | 13.05 | 10.59 | 10.06 | 15.99 | 8.00 | 3.00 |
| 40.00 | 19.92 | 19.76 | 19.51 | 19.04 | 18.00 | 12.99 |
| 50.00 | 21.69 | 23.81 | 22.66 | 22.87 | 21.00 | 15.05 |
| 60.00 | 22.81 | 24.66 | 27.15 | 25.21 | 19.00 | 22.90 |
| 70.00 | 24.17 | 23.83 | 30.14 | 30.79 | 19.00 | 22.12 |
| 80.00 | 24.99 | 22.41 | 28.98 | 35.13 | 20.00 | 30.00 |
| 90.00 | 24.30 | 20.95 | 25.14 | 30.96 | 18.00 | 27.00 |
| 100.00 | 21.76 | 19.58 | 21.98 | 22.00 | 18.00 | 27.00 |
| 110.00 | 17.90 | 18.12 | 19.83 | 20.00 | 18.00 | 27.00 |
| 120.00 | 13.86 | 16.29 | 14.63 | 20.00 | 18.00 | 21.00 |
| 130.00 | 10.52 | 13.90 | 14.00 | 20.00 | 16.00 | 21.00 |
| 140.00 | 7.04 | 11.00 | 14.00 | 18.00 | 16.00 | 21.00 |

|  | **Model** | | | | | |
| --- | --- | --- | --- | --- | --- | --- |
| 30.00 | 7.03 | 3.82 | 4.69 | 6.98 | 5.00 | 8.00 |
| 40.00 | 6.86 | 6.14 | 4.82 | 4.17 | 3.00 | 6.00 |
| 50.00 | 8.25 | 5.00 | 6.90 | 7.50 | 4.00 | 5.00 |
| 60.00 | 7.88 | 9.56 | 10.93 | 4.84 | 3.00 | 1.00 |
| 70.00 | 7.78 | 8.56 | 15.43 | 2.16 |  | 2.00 |
| 80.00 | 9.41 | 8.46 | 5.00 | 2.50 |  |  |
| 90.00 | 9.70 | 8.72 | 10.00 | 1.83 |  |  |
| 100.00 | 4.11 | 8.85 | 9.00 | 2.02 |  |  |
| 110.00 | 2.99 | 8.99 | 7.00 | 3.00 |  |  |
| 120.00 | 2.00 | 2.00 | 5.00 |  |  |  |
| 130.00 |  |  | 4.00 |  |  |  |
| 140.00 |  |  | 4.00 |  |  |  |
|  |  |  |  |  |  |  |
|  | **EXO** | | | | | |
| 30.00 | 5.00 | 4.00 | 4.00 | 5.00 | 5.00 | 4.00 |
| 40.00 | 11.00 | 3.00 | 5.00 | 5.00 | 6.00 | 4.00 |
| 50.00 | 10.00 | 1.00 | 6.00 | 2.00 |  | 5.00 |
| 60.00 | 8.00 | 4.00 | 7.00 | 3.00 |  | 3.00 |
| *Continued* | | | | | | |
| 70.00 | 6.00 | 4.00 | 6.00 |  |  | 10.00 |
| 80.00 | 5.00 | 4.00 | 6.00 |  |  | 10.00 |
| 90.00 | 4.00 | 3.00 | 6.00 |  |  | 8.00 |
| 100.00 | 5.00 | 3.00 | 5.00 |  |  | 10.00 |
| 110.00 | 5.00 | 3.00 | 5.00 |  |  |  |
| 120.00 | 5.00 |  | 4.00 |  |  |  |
| 130.00 | 5.00 |  | 4.00 |  |  |  |
| 140.00 | 4.00 |  | 4.00 |  |  |  |
|  |  |  |  |  |  |  |
|  | **NSC** | | | | | |
| 30.00 | 12.20 | 12.04 | 12.16 | 7.29 | 6.70 | 7.68 |
| 40.00 | 6.86 | 9.63 | 14.10 | 10.73 | 7.96 | 10.39 |
| 50.00 | 6.51 | 8.26 | 15.05 | 11.52 | 17.00 | 9.73 |
| 60.00 | 7.49 | 9.22 | 18.65 | 10.97 | 16.00 | 8.83 |
| 70.00 | 8.59 | 11.22 | 22.22 | 10.02 | 15.00 | 8.85 |
| 80.00 | 9.37 | 12.59 | 23.67 | 9.26 | 14.00 | 9.81 |
| 90.00 | 9.46 | 12.48 | 23.67 | 8.93 | 13.00 | 11.19 |
| 100.00 | 8.60 | 11.23 | 24.54 | 9.02 | 13.00 | 12.33 |
| 110.00 | 17.00 | 9.95 | 17.00 | 9.34 | 12.00 | 12.75 |
| 120.00 | 6.52 | 9.56 | 20.00 | 15.00 | 10.00 | 12.22 |
| 130.00 | 8.11 | 9.96 | 15.00 | 9.72 | 8.00 | 10.84 |
| 140.00 | 10.13 | 9.72 | 17.17 | 9.47 | 6.00 | 8.95 |
|  |  |  |  |  |  |  |
|  | **NSC+Exo** | | | | | |
| 30.00 | 5.70 | 7.31 | 10.22 | 10.00 | 8.02 | 8.72 |
| 40.00 | 8.30 | 8.33 | 16.84 | 7.00 | 12.92 | 9.10 |
| 50.00 | 7.09 | 13.27 | 19.45 | 11.00 | 16.89 | 5.07 |
| 60.00 | 7.49 | 18.89 | 21.29 | 16.00 | 17.13 | 5.58 |
| 70.00 | 10.22 | 23.37 | 23.66 | 20.00 | 18.03 | 10.07 |
| 80.00 | 13.15 | 25.89 | 26.71 | 24.00 | 22.32 | 14.47 |
| 90.00 | 13.86 | 26.32 | 29.98 | 25.00 | 18.00 | 15.45 |
| 100.00 | 11.52 | 24.96 | 22.00 | 24.00 | 11.00 | 12.48 |
| 110.00 | 7.59 | 22.35 | 24.00 | 22.00 | 11.00 | 7.80 |
| 120.00 | 4.90 | 19.09 | 24.00 | 20.00 | 11.00 | 4.68 |
| 130.00 | 5.25 | 15.74 | 23.00 | 19.00 | 7.00 | 4.96 |
| 140.00 | 5.95 | 12.76 | 21.00 | 19.00 |  | 7.12 |
