# Supplementary material for: NSC-derived exosomes enhance therapeutic effects of NSC transplantation on cerebral ischemia in mice
Source: eLife. 2023 Apr 27;12:e84493. doi: 10.7554/eLife.84493 (PMC10139690; doi:10.7554/eLife.84493)
Supplement: Figure 2—figure supplement 1—source data 1. [file elife-84493-fig2-figsupp1-data1.zip › Figure 2 supplement 1 source data 1/Figure 2 - supplement 1 source data 1.docx]

| *RBFOX3* | | | |
| --- | --- | --- | --- |
| **Sham** | 0.680272109 | 1.16691999 | 1.153711933 |
| **PBS** | 0.283763793 | 0.517423931 | 0.509850392 |
| **Exo** | 0.390288554 | 0.395540493 | 0.896756635 |
| **NSC** | 0.55089483 | 0.363218402 | 0.585887801 |
| **NSC+Exo** | 0.708361979 | 0.754824747 | 0.716401356 |

**Figure 2 - supplemant 1A-Resource data: qPCR:**

**Figure 2 - supplemant 1E-Resource data: qPCR:**

|  | **Ctrl** | | | **OGD/R** | | | **OGD/R+Exo** | | |
| --- | --- | --- | --- | --- | --- | --- | --- | --- | --- |
| *Bax* | 0.892857143 | 1.010330973 | 1.07546374 | 4.871270606 | 2.832628376 | 4.596248585 | 1.111553255 | 1.176252697 | 1.069769981 |
