# Supplementary material for: NSC-derived exosomes enhance therapeutic effects of NSC transplantation on cerebral ischemia in mice
Source: eLife. 2023 Apr 27;12:e84493. doi: 10.7554/eLife.84493 (PMC10139690; doi:10.7554/eLife.84493)
Supplement: Figure 3—source data 1. [file elife-84493-fig3-data1.zip › Figure 3-source data 1/Figure 3-source data 1.docx]

| NSC |  |  |  |  |  |  |  |  |  |  |  |  |  |
| --- | --- | --- | --- | --- | --- | --- | --- | --- | --- | --- | --- | --- | --- |
| Transplanted cell | 0.91 | | 1.09 | | 0.36 | | 0.55 | | 1.25 | | 0.58 | | 1.06 |
| Tuj-1% | 0.91 | | 1.10 | | 0.36 | | 0.55 | | 1.25 | | 0.58 | | 0.91 |
| Nestin% | 1.17 | | 0.83 | | 2.05 | | 0.45 | | 1.33 | | 2.77 | |  |
|  | |  |  |  |  |  |  |  |  |  |  |  |  |
| NSC+Exo |  |  |  |  |  |  |  |  |  |  |  |  |  |
| Transplanted cell | 1.30 | 1.26 | | 1.22 | | 1.33 | | 1.33 | | 1.30 | |  |  |
| Tuj-1% | 1.06 | 1.30 | | 1.26 | | 1.22 | | 1.33 | | 1.33 | |  |  |
| Nestin% | 0.89 | 0.46 | | 0.54 | | 0.60 | | 0.41 | | 0.46 | |  |  |

**Figure 3E-G-Resource data: Transplanted cell percentage:**
