# Supplementary material for: NSC-derived exosomes enhance therapeutic effects of NSC transplantation on cerebral ischemia in mice
Source: eLife. 2023 Apr 27;12:e84493. doi: 10.7554/eLife.84493 (PMC10139690; doi:10.7554/eLife.84493)
Supplement: Figure 4—source data 1. [file elife-84493-fig4-data1.zip › Figure 4-source data 1/Figure 4-source data 1.docx]

|  | **Ctrl** | | | **NSC** | | | **NSC+Exo** | | | |
| --- | --- | --- | --- | --- | --- | --- | --- | --- | --- | --- |
| CHOP | 0.28 | 1.00 | 0.85 | 1.50 | 2.31 | 1.39 | 0.77 | 1.19 | 0.68 |  |
| Nrf2 | 1.00 | 1.47 | 0.76 | 0.76 | 1.37 | 1.34 | 1.57 | 1.95 | 1.73 |  |
| NQO1 | 0.50 | 0.38 | 0.86 | 2.00 | 1.90 | 1.96 | 2.65 | 3.40 | 3.47 |  |
| Sod2 | 1.18 | 1.80 | 1.00 | 0.72 | 0.92 | 0.98 | 2.33 | 2.39 | 2.57 |  |

**Figure 4C-Resource data: qPCR:**

**Figure 4D-Resource data: MDA:**

| **Sham** | **Model** | **Exo** | **NSC** | **NSC+Exo** |
| --- | --- | --- | --- | --- |
| \| 3.17 \| \| --- \| | 7.95 | 7.00 | 8.18 | 7.92 |
| \| 3.60 \| \| --- \| | 7.90 | 6.18 | 8.25 | 7.78 |
| 3.10 | 9.20 | 5.93 | 8.23 | 7.36 |
| 3.73 | 9.50 |  |  |  |

**Figure 4E-Resource data: qPCR:**

| Gene | **Sham** | **Model** | **Exo** | **NSC** | **NSC+Exo** |
| --- | --- | --- | --- | --- | --- |
| TNF-a | 1.00 | 3.23 | 2.16 | 1.71 | 1.22 |
|  | 0.70 | 3.51 | 1.31 | 1.43 | 1.99 |
|  | 0.71 | 2.38 | 4.26 | 1.57 | 2.17 |
|  | 0.80 | 4.66 |  |  |  |
| IL-1b | 1.00 | 2.51 | 1.14 | 2.50 | 1.25 |
|  | 0.76 | 2.35 | 1.22 | 1.99 | 1.64 |
|  | 1.75 | 3.27 | 2.77 | 1.96 | 1.89 |
|  | 0.96 | 1.85 |  |  |  |
| IL-10 | 2.59 | 0.08 | 1.66 | 2.04 | 3.01 |
|  | 1.00 | 0.44 | 1.12 | 0.75 | 2.10 |
|  | 0.90 | 0.20 | 2.10 | 0.17 | 2.19 |

**Figure 4H-Resource data: Glial scar area:**

| sham | model | Exo | NSC | NSC+Exo |
| --- | --- | --- | --- | --- |
| 0.52 | 38.86 | 15.99 | 6.99 | 2.82 |
| 1.48 | 38.72 | 22.04 | 26.88 | 4.60 |
|  | 37.18 | 25.59 | 15.64 | 0.53 |
|  |  |  | 15.16 |  |
