# Supplementary material for: NSC-derived exosomes enhance therapeutic effects of NSC transplantation on cerebral ischemia in mice
Source: eLife. 2023 Apr 27;12:e84493. doi: 10.7554/eLife.84493 (PMC10139690; doi:10.7554/eLife.84493)
Supplement: Figure 4—figure supplement 1—source data 1. [file elife-84493-fig4-figsupp1-data1.zip › Figure 4 supplement 1 source data 1/Figure 4 - supplement 1 source data 1.docx]

**Figure 2 - supplemant 1B-Resource data: qPCR:**

| Astrocytes  -24h | **Ctrl** | | | **OGD/R** | | | **OGD/R+Exo** | | |
| --- | --- | --- | --- | --- | --- | --- | --- | --- | --- |
| *C3* | 0.950329938 | 0.645692777 | 1.286479643 | 8.480558829 | 14.30150484 | 7.537019591 | 4.37684991 | 3.525035801 | 4.37684991 |
|  | 2.023237822 | 0.80072007 | 0.294110355 | 8.610357479 | 16.42473555 | 8.157766488 | 3.203065788 | 2.196158066 | 3.203065788 |
| *Gbp2* | 1.039929897 | 0.801100951 | 1.300331273 | 2.093395765 | 5.087689094 | 3.705176578 | 0.780125617 | 1.074217308 | 0.52251638 |
|  | 0.935585275 | 0.624475446 | 1.300331273 | 1.380283058 | 3.158951915 | 3.056958015 | 0.84990384 | 0.99826993 | 0.65701358 |
| *Lcn2* | 0.980462792 | 0.653134531 | 1.432345063 | 0.884322457 | 1.022585159 | 1.325742378 | 0.809031434 | 0.451971616 | 0.778303801 |
|  | 1.215866529 | 0.729326675 | 0.988639692 | 0.974624545 | 0.920350355 | 1.291304332 | 0.738126802 | 0.355661734 | 0.90582135 |

**Figure 2 - supplemant 1C-Resource data: qPCR:**

| Astrocytes  -48h | **Ctrl** | | | **OGD/R** | | | **OGD/R+Exo** | | |
| --- | --- | --- | --- | --- | --- | --- | --- | --- | --- |
| *C3* | 1.066136129 | 0.713336454 | 1.219514645 | 2.493039291 | 3.185840913 | 2.610298396 | 2.223009162 | 2.308941814 | 2.487024023 |
|  | 0.599721802 | 0.605525969 | 1.00434375 | 2.019175633 | 4.158495644 | 1.90747841 | 1.916191224 | 1.457955142 | 1.617754178 |
| *Gbp2* | 0.70289616 | 1.492387455 | 1.066238265 | 0.740109593 | 1.382017838 | 1.118572755 | 1.050283351 | 1.33817699 | 0.661875989 |
|  | 1.027619541 | 0.828778283 | 0.884321146 | 0.578574002 | 1.254707484 | 1.535432156 | 1.118101052 | 0.782713856 | 0.896695811 |
| *Lcn2* | 0.470253768 | 1.322067181 | 1.218491831 | 2.738135537 | 3.738587755 | 6.834466497 | 0.817650626 | 2.033752615 | 1.500505303 |
|  | 0.413594029 | 1.629189755 | 0.945980069 | 2.636644654 | 2.519775828 | 7.825000968 | 0.897545173 | 1.728378894 | 1.128308655 |
