# Supplementary material for: NSC-derived exosomes enhance therapeutic effects of NSC transplantation on cerebral ischemia in mice
Source: eLife. 2023 Apr 27;12:e84493. doi: 10.7554/eLife.84493 (PMC10139690; doi:10.7554/eLife.84493)
Supplement: Figure 5—figure supplement 1—source data 1. [file elife-84493-fig5-figsupp1-data1.zip › Figure 5 supplement 1-source data 1/Figure 5 supplement 1-source data 1.docx]

| miRNA | Ct1 | Ct2 | Ct3 |
| --- | --- | --- | --- |
| hsa-miR-25-3p | 26.31 | 26.23 | 26.15 |
| hsa-let-7i-5p | 24.02 | 24.25 | 24.18 |
| hsa-miR-30a-5p | 27.14 | 28 | 27.07 |
| hsa-miR-218-5p | 22.98 | 23.31 | 23.98 |
| hsa-miR-151a-3p | 27.77 | 27.13 | 27.09 |
| hsa-miR-21-5p | 23.46 | 23.34 | 23.58 |
| hsa-miR-26a-5p | 22.04 | 22.31 | 22.72 |
| hsa-miR-148a-3p | 19.57 | 19.66 | 19.6 |
| hsa-miR-7-5p | 17.45 | 17.27 | 17.32 |
| hsa-miR-9-5p | 15.97 | 15.02 | 15.34 |

**Figure 5 supplement 1A-Resource data: q PCR of miRNA:**

**Figure 5 supplement 1B-Resource data: q PCR of gene:**

|  | **Ctrl** | | | **NSC** | | | **NSC+Exo** | | |
| --- | --- | --- | --- | --- | --- | --- | --- | --- | --- |
| STAT3 | 0.83 | 1.17 | 1 | 2.82 | 3.05 | 2.14 | 0.67 | 1.30 | 0.31 |
| PTPN1 | 1 | 1.43 | 0.79 | 3.26 | 2.82 | 2.37 | 0.33 | 0.8 | 1.28 |
| CHUK | 0.97 | 1.25 | 1 | 3.17 | 3.31 | 3.46 | 0.2 | 0.3 | 1.71 |
